# Variation in sperm performance and mitochondrial metabolism of *Mytilus* spp. from the North and Baltic Seas under different environmental scenarios

**DOI:** 10.1242/jeb.251452

**Published:** 2026-02-10

**Authors:** Hui Kong, Inna M. Sokolova

**Affiliations:** ^1^ Department of Marine Biology, Institute for Biological Sciences, University of Rostock, 18059 Rostock, Germany; ^2^ Department of Maritime Systems, Interdisciplinary Faculty, University of Rostock, 18059 Rostock, Germany

**Keywords:** *Mytilus edulis*, Sperm motility, Mitochondrial function, ROS production, Osmolarity, Fertilization

## Abstract

Climate change, including seawater warming and salinity fluctuations, is increasingly affecting marine ecosystems worldwide. The blue mussel, *Mytilus edulis*, widely distributed along the temperate coasts of the Northern Hemisphere, thrives in environments characterized by temperature fluctuations and salinity gradients. In particular, populations in the Baltic and North Seas are exposed to significant variation in these factors, which can affect the reproductive capacity of blue mussels, essential for sustainability of their populations. This study assessed the effects of varying temperature and salinity on the reproductive performance of blue mussels from the Baltic and North Seas, focusing on sperm motility, ATP content and fertilization success. Additionally, sperm mitochondrial function in Baltic Sea mussels was examined under different temperature and osmolarity conditions. The results showed that mussels from both populations tolerated seawater warming, but were sensitive to cold and low salinity, with sperm motility and fertilization success significantly impaired under these conditions. The salinity window for sperm motility and fertilization was population specific: optimal ranges were a salinity of 13–17 for Baltic Sea mussels and 21–35 for North Sea mussels. Notably, North Sea mussels were unable to reproduce at salinity 9, whereas Baltic Sea mussels were severely impaired at salinity 5. High temperature (25°C) reduced mitochondrial respiratory efficiency and increased reactive oxygen species (ROS) production, while osmolarity did not appear to be a key factor. These findings highlight population-specific reproductive traits in *M. edulis* and link sperm performance to mitochondrial function, providing new insights into benthic adaptation to changing coastal environments.

## Teaser

**Summary:** Salinity and temperature impact the sperm performance of North and Baltic Sea *Mytilus edulis*, with optimal conditions reflecting the specific environmental characteristics of their distinct native habitats.

## INTRODUCTION

Reproduction is a fundamental biological process that ensures the continuity of species through the production of offspring (Fusco and Minelli, 2019). In marine bivalves, such as mussels, environmental factors such as temperature and salinity play a crucial role in reproduction (Boroda et al., 2020; Lymbery et al., 2021; Tackett et al., 2024). As broadcast spawners, marine bivalves release their gametes directly into the seawater for external fertilization, making them particularly vulnerable to environmental stressors (Boni et al., 2016; Leite et al., 2024; Parker et al., 2009).

Climate change poses significant challenges to marine ecosystems, with rising seawater temperatures and increased frequency of marine heatwaves (Fernández et al., 2024; Frölicher et al., 2018; Hobday et al., 2016). In addition, extreme weather events, such as storms, storm surges, flooding, heavy precipitation and droughts, lead to changes in seawater salinity, particularly in semi-enclosed seas such as the Baltic Sea (Lehmann et al., 2022; Meier et al., 2022; Rutgersson et al., 2022). These temperature and salinity fluctuations have been shown to negatively affect the reproductive capacity of marine bivalves such as mussels and oysters (Eads et al., 2016; Gregory et al., 2023). For instance, increased temperature and reduced salinity have been linked to reduced sperm survival in *Crassostrea gigas* (Cheng et al., 2024), as well as decreased fertilization success in *C. gigas* exposed to hyposaline waters (Mooney, 2016). In mussels, *Mytilus galloprovincialis*, thermal stress has been associated with reduced sperm concentration, mitochondrial membrane potential collapse, increased lipid peroxidation, DNA fragmentation and morphological abnormalities (Boni et al., 2016). Consequently, warming seawater and fluctuating salinity may disrupt the reproductive success of coastal bivalves, potentially leading to shifts in population dynamics and marine ecosystem structure. However, little is known about how temperature and salinity fluctuations affect the mitochondrial function of bivalve sperm, which is a key driver of motility and fertilization success. Moreover, population-specific differences in reproductive physiology between North Sea and Baltic Sea blue mussels, *Mytilus edulis*, remain poorly understood.

*Mytilus edulis*, which is widely distributed along the northern Atlantic coasts, demonstrates significant physiological plasticity, reflecting adaptations to variable environmental conditions (Larsson et al., 2017). The thermal tolerance window of adult *M. edulis* spans from 0 to 29°C with an optimum temperature around 20°C (Larsson et al., 2017; Seuront et al., 2019; Sorte et al., 2019), and both the North Sea and Baltic Sea serve as natural habitats for the species, suggesting broad salinity tolerance (Kautsky, 1982; Sprung, 1983). However, low salinity limits local population structure and growth rates, particularly in the Baltic Sea, where populations exhibit smaller sizes, thinner shells and slower growth compared with their North Sea counterparts (Kautsky et al., 1990; Westerbom et al., 2002). Despite these known physiological adaptations, little is understood about the reproductive strategies of these populations, particularly in relation to sperm performance and fertilization under varying salinity and temperature conditions.

In marine bivalves, oxidative phosphorylation (OXPHOS) in sperm provides the energy necessary for motility via aerobic ATP production in the mitochondria, a process that is highly oxygen dependent (Kong and Sokolova, 2024). However, mitochondrial responses to environmental stressors, such as thermal stress, vary among male gametes of marine invertebrate species. For example, thermal stress (at 28°C) in *M. galloprovincialis* induces a collapse in mitochondrial membrane potential of the sperm (Boni et al., 2016), while no such effect has been observed in the sea urchin *Heliocidaris tuberculata* (at 20–26°C), which showed no correlation between sperm mitochondrial activity and fertilization success (Binet and Doyle, 2013). While climate change studies on sperm performance have largely focused on motility, viability and fertilization success, little is known about how environmental stressors affect mitochondrial function in sperm and the relationship between mitochondrial activity and sperm performance in marine bivalves.

This study examined how temperature and salinity affect the reproductive performance of *M. edulis*, comparing gamete quality, fertilization success and embryonic development between North Sea and Baltic Sea populations. In addition, we investigated how environmental stressors influence sperm mitochondrial activity and its relationship with sperm performance, thereby assessing potential population-specific sensitivities to changing coastal conditions. We hypothesized that warming and low salinity will reduce mitochondrial efficiency and increase ROS production in mussel sperm. Additionally, we expected sperm from Baltic Sea mussels to perform better in low salinity than those from the North Sea, as they are more adapted to brackish conditions. Lastly, we propose that temperature and salinity fluctuations will significantly affect sperm performance, fertilization success and embryonic development in both populations.

## MATERIALS AND METHODS

### Animal collection and maintenance

Mussels, *Mytilus edulis* Linnaeus 1758, of the Baltic Sea population were collected from Warnemünde (54°10′49.6″N 12°05′21.5″E), Rostock, Germany, in February 2023 and 2024 (300 individuals for each collection), because of the limited reproductive window and availability of ripe gametes. The temperature exposure for the Baltic Sea mussel population was conducted in 2023; the salinity exposure and sperm mitochondrial experiments for this population were completed in 2024. The North Sea mussel population was collected from Sylt (55°01′18.6″N 8°26′25.9″E), Germany, in March 2023. In both study regions, the timing of our collections shortly preceded the seasonal peak of spawning in order to obtain ready-to-spawn individuals with mature gametes. In the Baltic Sea, mussels typically undergo rapid gamete maturation at the beginning of March (Kautsky, 1982), whereas in the North Sea, most individuals reach full maturity by February (Sprung, 1983). A total of 300 adult mussels (4–6 cm) were immediately transported dry by car at ambient temperature, arriving at the laboratory within 12 h. The mussels were kept at 10°C for at least 2 weeks in two independent recycling aerated systems with a salinity of 30 (practical salinity units) for the North Sea mussels and a salinity of 15 for the Baltic Sea mussels. The chosen acclimation salinities were similar to the respective habitat salinities of the North Sea (salinity 30–32) and Baltic Sea (salinity 16) populations (Rick et al., 2022; Kong and Sokolova, 2025). Salinity values for both maintenance systems were monitored using a conductivity-based multimeter (Hach HQ40d) and adjusted as needed. Each system had 12 flow-through tanks with a total volume of 570 l of seawater. To prepare the seawater, 20% natural seawater from Warnemünde was mixed with 80% artificial seawater (Tropic Marin^®^ Pro-Reef, Tropic Marin AG, Hünenberg, Switzerland) to achieve different salinity levels. The mixture was aerated for 3 days before use. A feeding device (Reefdoser EVO 4, Aqua Medic) continuously added microalgae mixture to the maintenance systems at a rate of 20 ml h^−1^. The mixture consisted of live *Nannochloropsis* sp., live *Rhodomonas salina* and a commercial shellfish diet (DT's Live Marine Phytoplankton, Coralsands, Wiesbaden, Germany) at a ratio of 1:1:1. Chlorophyll concentration in seawater was routinely monitored to ensure sufficient food for mussels.

The two studied populations of *Mytilus* spp. possess a different genetic background. Specifically, the Warnemünde population (Baltic Sea) originates from a stable hybrid zone between *M. edulis* and *Mytilus trossulus*, with ∼70% of nuclear markers and 100% of mitochondrial markers of *M. edulis* (Stuckas et al., 2017), and is thus classified as a natural hybrid of *M. edulis* (and hereafter referred to as *M. edulis*). In contrast, the Sylt population (North Sea) is genetically pure *M. edulis*, according to prior population genetic assessments (Knöbel et al., 2020). It is important to note that all fertilization experiments in the present study were conducted using eggs and sperm of the mussels belonging to the same population.

### Mussel spawning and fertilization

A thermal shock method was used to induce the spawning of mussels as previously described (Kong and Sokolova, 2024; Ramesh et al., 2017). Briefly, the adult mussels were placed individually in beakers filled with 250 ml of 0.2 μm filtered artificial seawater (FSW). Salinity of the FSW was 30 and 15 for the North Sea mussels and the Baltic Sea mussels, respectively. Temperature was gradually increased from 10°C to 23°C within 20 min in a water bath. Generally, gamete release started 20–30 min after thermal shock initiation. Gametes were collected approximately 1 h after the onset of spawning. Complete gamete release was confirmed visually by the cessation of the spawning stream. After that, the adult mussels were removed from the beakers, and the remaining seawater containing the gametes was gently swirled. For mitochondrial analysis, the sperm suspension was centrifuged to collect sperm, while for motility and fertilization assays, aliquots of the fresh suspension were used immediately. This thermal shock procedure resulted in a spawning success rate of approximately 50–60% across both populations. The sperm concentration was determined with a hemocytometer (M-NZ-4CH, Kisker Biotech). Egg concentration was measured by first removing the adult mussels from the beaker containing 250 ml of seawater, where eggs had settled. The seawater was gently swirled to evenly distribute the eggs without causing mechanical stress, which could compromise egg quality. A 50 µl sample of this homogenized suspension was transferred into 950 µl of water in a 2 ml Eppendorf tube, achieving a 20-fold dilution. After mixing the diluted sample well, a 50 µl subsample was placed on a concave slide for microscopic examination (Olympus, CH3-TR45). Egg counting was performed three times (*n*=3), with the concentration calculated as the average count multiplied by 400 (eggs ml^−1^). The final egg concentration for sperm to egg ratio calculation for fertilization was the mean of these three measurements. For fertilization, active sperm and eggs were gently mixed at a 200:1 sperm:egg ratio and left undisturbed for 2 h (Bechmann et al., 2011; Pechenik et al., 1990; Tidau et al., 2023). Fertilization was conducted at approximately 2.5 h post-exposure. In the salinity experiment, fertilization for all treatments was carried out at 15°C. In contrast, in the temperature experiment, fertilization was performed at the respective exposure temperature of each treatment.

For each fertilization trial, eggs and sperm were collected from a single female and a single male, respectively, from the corresponding population (North Sea or Baltic Sea), to minimize inter-individual variability in gamete quality. The first polar body release and the zygote cleavage were used as markers of fertilization success (Sprung and Bayne, 1984; Ventura et al., 2016). The fertilization success rate (FSR) was calculated as the percentage of fertilized eggs out of the total number of eggs used in the fertilization trials.

### Experimental design

#### Experiment 1: effects of temperature and salinity on the reproductive traits of Baltic Sea versus North Sea mussels

To examine the effects of temperature and salinity on reproductive performance, we assessed sperm motility and ATP content, fertilization success and embryonic development in blue mussels, *M. edulis*, from the North Sea and Baltic Sea. Fresh sperm and egg suspensions were collected 1 h after spawning, induced by thermal shock, and divided into five aliquots (40 ml for sperm and 30 ml for eggs) before exposure to the five temperature or salinity treatments. The spawning medium salinity was set to 15 for Baltic Sea mussels and 30 for North Sea mussels. Temperature and salinity exposures were carried out in separate experimental runs.

In the salinity exposure experiment, sperm and eggs were subjected to one of five salinities spanning a habitat-specific salinity range. Salinity in each of the five aliquots of sperm or egg suspension was adjusted by gradually adding distilled water or sea salt (Tropic Marin^®^ Pro-Reef, Tropic Marin AG) under constant gentle mixing, with five biological replicates for all salinity treatments (*n*=5 per sex and population). Gametes from the Baltic Sea mussels were exposed to salinities of 5, 9, 13, 17 and 21, while those from the North Sea mussels were exposed to salinities of 9, 17, 21, 30 and 35 (Fig. S2). The test salinity ranges were selected to reflect typical habitat salinities (9–15 for Baltic Sea and 28–35 for North Sea populations) and laboratory acclimation salinities (15 for Baltic Sea and 30 for North Sea mussels). To enable cross-population comparisons, three common salinities (9, 17 and 21) were included in the experimental design. Fertilization experiments were initiated 2.5 h after the target salinities were achieved. Sperm suspension was gently added to 30 ml of FSW containing eggs to achieve a sperm to egg ratio of 200:1, followed by thorough mixing. Fertilization success was assessed by counting zygotes, embryos and unfertilized eggs in photographs taken under a microscope (Olympus, CH3-TR45) 2 h post-fertilization. The sample size for the fertilization experiments was 5, with each family derived from gametes of different males and females. The final salinity of each experimental sample, including gamete suspensions and fertilization media, was measured using a multimeter (Hach, HQ40d) after exposure.

To evaluate the effect of salinity on sperm performance, sperm suspensions exposed to different salinities were maintained for 4 h post-spawning at 15°C. Sperm performance metrics, including motility, velocity curvilinear (VCL) and velocity average path (VAP), were assessed at the spawning salinity (15 and 30 for Baltic Sea and North Sea populations, respectively) immediately prior to salinity adjustment (time 0) and after 1 and 4 h of salinity exposure. Sperm movement videos were recorded using a microscope (Olympus CH3-TR45) equipped with a camera connected to the software ToupView on a computer. Videos were captured at 30 frames s^−1^ for 60 s, with three replicate videos taken for each sperm suspension sample. Video analysis was performed using the computer-assisted sperm analysis (CASA) plugin for ImageJ (https://imagej.net/software/imagej/#publication), adapted for mussel sperm (Kong and Sokolova, 2024; Shi et al., 2017; Wilson-Leedy and Ingermann, 2007). Additionally, sperm ATP content was measured 3 h post-exposure using the CellTiter-Glo^®^ 2.0 cell viability assay kit (Promega, Walldorf, Germany) with a SpectraMax ID3 Multi-Mode Microplate Reader (Molecular Devices), following the protocol previously described by Kong and Sokolova (2024). Sperm samples from 5 males in each population were analyzed (*n*=5 for all treatments).

In the temperature exposure experiment, gametes (sperm or eggs) were collected from five individual mussels per sex for each population (*n*=5 per sex and population), except for the sperm samples from the Baltic Sea mussels (*n*=6). Each gamete sample was evenly divided into five aliquots, which were then exposed to five temperature conditions (5, 10, 15, 20 and 25°C) in a water bath (Fig. S2). Salinity was maintained at 15 for Baltic Sea gametes and 30 for North Sea gametes across all temperature treatments. The temperature range was selected to represent environmental conditions from early spring, when occasional spawning might occur at low temperatures (around 5°C), through the main reproductive season (April–June, 10–15°C), and including elevated temperatures (20–25°C) associated with spring and summer heat waves. In the Baltic Sea, seawater salinity generally remains low and stable throughout the year, while temperatures increase gradually from early spring (∼5°C) to late spring/early summer (∼15°C), with occasional heatwaves reaching up to ∼25°C (Naumann et al., 2018; Rutgersson et al., 2022). In contrast, the North Sea shows higher baseline salinity with distinct seasonal temperature dynamics and occasional heatwaves. These temporal dynamics were considered when selecting the experimental temperature range to reflect both gradual seasonal changes and episodic extreme events.

After 4 h of exposure to the respective temperatures, the same endpoints – sperm motility, ATP content, fertilization success and embryonic development – were measured as in the salinity exposure experiment. The detailed timing and sequence of these measurements are illustrated in Fig. S1. The temperature of each treatment, for both sperm suspension and fertilization samples, was controlled using automated water baths (Thermo Haake K10, Thermo Haake SC100, Thermo Haake D1, Biometra KH-3 and Fisherbrand FBC620). Temperature was recorded with a digital thermometer (EBI 20-TE1) before and after the exposure to ensure accuracy.

The temperature and salinity conditions for all experimental exposures are shown in Table S1.

#### Experiment 2: combined effects of temperature and osmolarity on sperm mitochondrial function

Mitochondrial function in sperm from Baltic Sea males was assessed using a high-resolution respirometer (Oxygraph 2-k, Oroboros Instruments, Innsbruck, Austria) equipped with integrated software (DatLab 6). Sperm suspension samples (50 ml) were collected from individual males 1 h post-spawning and centrifuged at 8000 ***g*** for 2 min. The resulting sperm pellet was resuspended in 500 μl of ice-cold homogenization buffer (30 mmol l^−1^ Hepes pH 7.5, 100 mmol l^−1^ sucrose, 200 mmol l^−1^ KCl, 100 mmol l^−1^ NaCl, 8 mmol l^−1^ EGTA, 30 mmol l^−1^ taurine) supplemented with freshly added protease inhibitors (1 mmol l^−1^ phenylmethanesulfonyl fluoride and 2 μg ml^−1^ aprotinin). The sperm suspension was injected (200 μl per chamber) into the temperature-controlled chambers of the Oxygraph 2-k for measurement. Sperm concentration of both raw and resuspended samples was determined using a hemocytometer (Table S2).

Sperm mitochondrial function was assessed across three temperatures (15, 20 and 25°C) and five osmolarities (630, 510, 390, 270 and 150 mOsm) that mimic seawater osmolarity at salinities of 21, 17, 13, 9 and 5, respectively (Santos et al., 2013; Thurman et al., 2013). A fully crossed temperature×osmolarity design was used (*n*=5–8 per treatment; Fig. S2), with assay buffer compositions adjusted for each osmolarity (Table S3).

Calibration for oxygen and ROS sensors was conducted following established protocols (Ouillon et al., 2021; Steffen et al., 2023). ROS efflux was measured using the Fluorescence-Sensor Green (525 nm) integrated with the Oxygraph 2-k. Hydrogen peroxide (H_2_O_2_) production was quantified using 10 μmol l^−1^ Amplex™ UltraRed (AMR) as a reporter, 1 U ml^−1^ horseradish peroxidase to catalyze H_2_O_2_-dependent AMR conversion, and 5 U ml^−1^ superoxide dismutase to convert superoxide radicals to H_2_O_2_. Fluorometric sensors were calibrated with 0.2 μmol l^−1^ H_2_O_2_ prior to measurements.

For each measurement, 200 μl of sperm suspension was injected into the 2 ml chambers following calibration. Digitonin (10 μg ml^−1^) was added to permeabilize sperm membranes, and real-time monitoring of oxygen consumption and ROS efflux was performed. Mitochondrial respiration and ROS efflux were evaluated in permeabilized sperm by measuring these parameters in the LEAK state (state 2) after adding 5 mmol l^−1^ pyruvate, 1 mmol l^−1^ malate and 10 mmol l^−1^ succinate to stimulate electron flux through Complexes I and II, and in the OXPHOS state (state 3) following the addition of 3.75 mmol l^−1^ ADP to activate F_o_,F_1_-ATP synthase. Respiration rate in the LEAK state indicates the oxygen consumption required to maintain mitochondrial membrane potential without ATP production, reflecting the cost of compensating for futile proton and cation cycling (Jastroch et al., 2010; Sokolova, 2023). OXPHOS state (state 3) respiration served as a proxy for the maximum ATP synthesis capacity of mitochondria (Sokolova, 2023). The respiratory control ratio (RCR), a measure of mitochondrial coupling efficiency, was calculated as the ratio of OXPHOS to LEAK respiration (state 3 to state 2) (Estabrook, 1967). Fractional electron leak (FEL) was determined as the ratio of H_2_O_2_ efflux to oxygen consumption (*Ṁ*_O_2__) during both LEAK and OXPHOS states, based on simultaneous measurement of mitochondrial oxygen consumption and ROS efflux.

### Statistical analysis

SPSS version 23.0 (IBM Corp., Armonk, NY, USA) was used for statistical analysis, and GraphPad Prism version 8.3 (GraphPad Software Inc., La Jolla, CA, USA) was used to generate figures. Shapiro–Wilk's test was applied to assess the normality of data distribution, and Levene's test was used to evaluate homogeneity of variance. Percentage data were arcsine square-root transformed to meet assumptions of normality and homogeneity where required. For experiment 1, two-way repeated measures ANOVA was conducted to examine interactions between temperature or salinity and exposure time on response variables, including sperm motility, VCL and VAP. The effects of temperature or salinity were further analyzed using one-way ANOVA followed by Tukey's *post hoc* test for normally distributed data, or the Kruskal–Wallis test for non-normally distributed data or datasets violating homogeneity of variance. The effects of exposure time were analyzed using one-way repeated measures ANOVA for normally distributed data or Friedman's ANOVA for non-normally distributed data.

For experiment 2, one-way ANOVA followed by Tukey's *post hoc* test was used for normally distributed data, while the Kruskal–Wallis test was applied for non-normally distributed data. Welch's ANOVA followed by Games–Howell *post hoc* test was used for normally distributed data with unequal variances. Two-way ANOVA was not performed because of non-normal distribution in parts of the dataset. Pearson's and Spearman's correlation were conducted to evaluate relationships among sperm motility, ATP, fertilization success and mitochondrial function, based on normality of the variables. All data are presented as means±s.e.m. Statistical significance was set at *P*<0.05.

## RESULTS

### Effects of temperature and salinity on the reproduction of mussels from the Baltic versus North Sea

#### Sperm motility

**Fig. 1.**
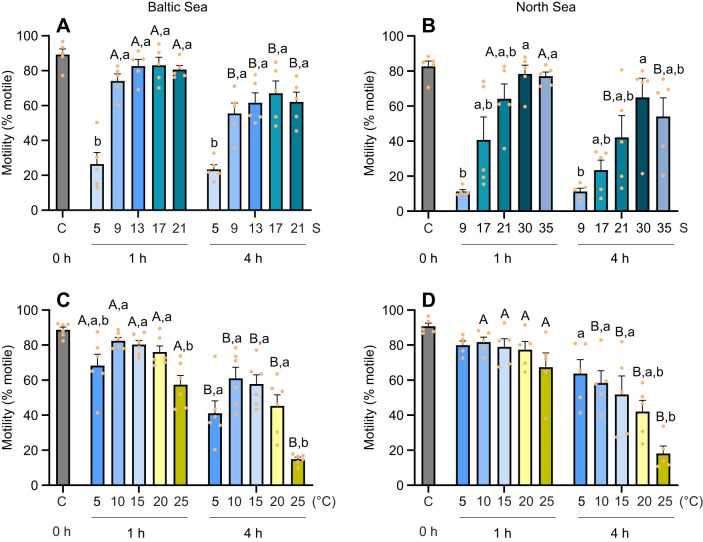
**Motility of sperm exposed to different temperature or salinity conditions, for Baltic Sea and North Sea blue mussel, *Mytilus edulis*, populations.** Sperm motility of Baltic Sea (left) and North Sea (right) blue mussels, 1 and 4 h after exposure to different salinity (A, *n*=5; B, *n*=5) or temperature (C, *n*=6; D, *n*=5) treatments. The 0 h time point (control, C) in all graphs represents sperm measurements immediately prior to salinity and temperature exposure, taken at the acclimation conditions (15°C, salinity 15 for the Baltic Sea mussels and salinity 30 for the North Sea mussels). Means+s.e.m.; data points represent individual biological replicates. Different capital letters represent significant differences between the 1 h and 4 h time points (*P*<0.05). Different lowercase letters indicate significant differences among the five temperature/salinity treatment groups within the same time point (*P*<0.05). Data were analyzed using two-way repeated measures ANOVA to examine interactions. For comparisons among groups, one-way ANOVA with Tukey's *post hoc* test or Kruskal-Wallis test was applied depending on data normality and homogeneity. See Materials and Methods, ‘Statistical analysis’ for details.

The percentage of motile sperm from the Baltic Sea population remained high and stable in relatively high-salinity seawater environments (salinity 13, 17 and 21; approximately 80% motile sperm), compared with the low-salinity seawater environment (salinity 5, with only 26.5±6.7% and 23.5±2.7% motile sperm at 1 h and 4 h post-exposure, respectively, significantly lower than in the other treatments). At salinity 9 (close to the average habitat salinity of the Baltic Sea population), 74.1±3.9% of Baltic Sea mussel sperm were motile. A significant decrease in the percentage of motile sperm was observed in all salinity treatments, except for salinity 5, after 4 h post-exposure compared with 1 h (Fig. 1A).

The percentage of motile sperm from the North Sea population was slightly lower than that of the Baltic Sea population immediately after spawning (time 0), with 82.6±3.1% motile sperm from North Sea mussels and 89.2±3.3% from Baltic Sea mussels (Fig. 1A,B). Sperm from the North Sea population showed much less tolerance to low-salinity environments. Only 11.2±1.1% and 11.2±1.9% of sperm were motile at 1 h and 4 h post-exposure to salinity 9, respectively. At salinity 17, 40.7±13.1% of sperm from the North Sea population were active after 1 h exposure, compared with 83.1±4.6% from the Baltic Sea population. Additionally, 64.1±8.5% of North Sea mussel sperm were motile at 1 h post-exposure in salinity 21, but this decreased significantly to 42.1±12.4% after 4 h. The optimal salinity for North Sea mussel sperm motility was 30, where 78.4±5.0% of sperm were motile after 1 h, and no significant reduction was observed after 4 h. In contrast, at salinity 35, sperm motility significantly decreased after 4 h, with 77.1±2.4% motile at 1 h post-exposure, dropping to 54.0±10.7% after 4 h (Fig. 1B).

Sperm from both populations showed tolerance to temperatures ranging from 5°C to 20°C, maintaining a high percentage of motile sperm during the first hour after spawning. However, at 25°C, a decrease in sperm motility of the Baltic Sea population was observed at this time point (Fig. 1C). After 4 h of exposure, sperm motility declined in all temperature conditions, except for the North Sea population at 5°C (Fig. 1C,D). The Baltic Sea mussel sperm appeared to be less tolerant to extreme temperatures compared with those of the North Sea population. In the Baltic Sea population, sperm motility decreased after 4 h at 5°C, while it remained stable in the North Sea population. After 4 h at 25°C, sperm motility was reduced to 18.1±4.2% in the North Sea population and 14.9±1.2% in the Baltic Sea population (Fig. 1C,D). Besides, significant interactions between exposure time and temperature were observed in the motility rate of sperm from both Baltic Sea and North Sea populations (Table S5).

#### Sperm velocity and ATP content

**Fig. 2.**
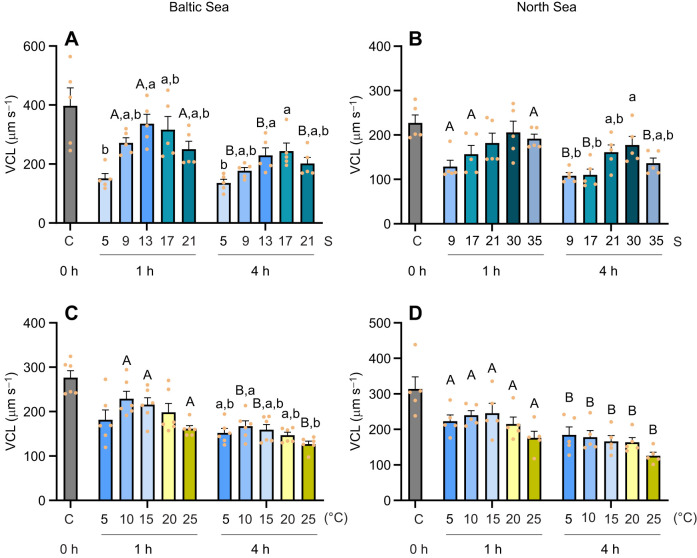
**Velocity curvilinear (VCL) of sperm exposed to different temperature or salinity conditions, for Baltic Sea and North Sea *M. edulis* populations.** VCL of sperm from Baltic Sea (left) and North Sea (right) blue mussels, 1 and 4 h after exposure to different salinity (A, *n*=5; B, *n*=5) or temperature (C, *n*=6; D, *n*=5) treatments. Means±s.e.m.; data points represent individual biological replicates. Different capital letters represent significant differences between 1 h and 4 h time points (*P*<0.05). Different lowercase letters indicate significant differences among the five temperature/salinity treatment groups within the same time point (*P*<0.05). Statistical tests are detailed in Fig. 1.

The sperm VCL of Baltic Sea mussels remained relatively stable after 1 h in the salinity range 9–21, with a significant decline observed at salinity 5. The highest sperm VCL values (316–337 μm s^−1^) were recorded at salinity 13 and 17 in the Baltic Sea population. In contrast, the average VCL in other treatments remained below 300 μm s^−1^, with the lowest values observed at salinity 5 (151.2±16.8 μm s^−1^). Furthermore, after 4 h, VCL in Baltic Sea mussel sperm decreased at salinities greater than 5 (with significant reductions at salinity 9, 13 and 21) compared with 1 h post-exposure (Fig. 2A). At salinity 5, sperm VCL remained at low levels, similar to 1 h post-exposure. In North Sea mussels, sperm VCL remained robust across all salinity treatments (9–35) after 1 h of exposure. However, after 4 h, VCL significantly decreased at salinity 9, 17 and 35, with VCL in the salinity 30 treatment being significantly higher than that in both the 9 and 17 treatments (Fig. 2B).

Sperm VCL in both the Baltic Sea and North Sea populations was resilient to seawater temperature variations. Only after 4 h at 25°C did VCL of the Baltic Sea mussel sperm significantly decrease compared with the 10°C treatment. Over time, sperm VCL in all five temperature treatments slowed in the North Sea population, while in the Baltic Sea population, significant effects of exposure duration were observed at 10, 15 and 25°C (Fig. 2C,D). However, no significant interaction was observed between exposure time and salinity/temperature on sperm VCL in both Baltic Sea and North Sea populations (Table S5).

**Fig. 3.**
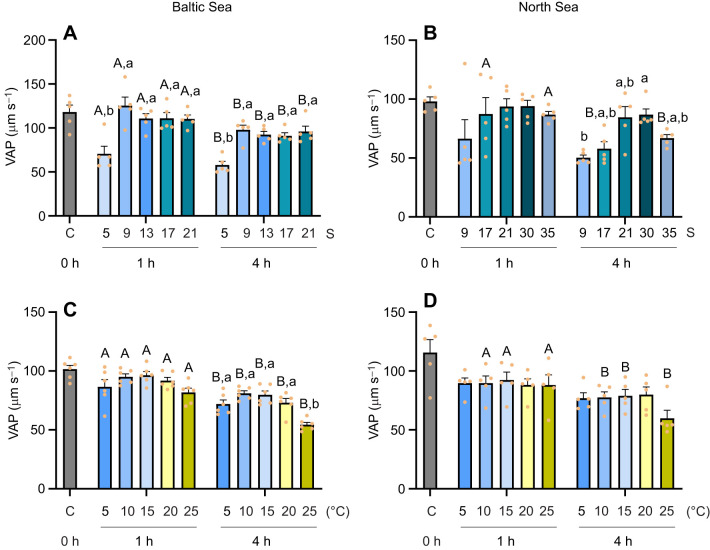
**Velocity average path (VAP) of sperm exposed to different temperature or salinity conditions, for Baltic Sea and North Sea *M. edulis* populations.** VAP of sperm from Baltic Sea (left) and North Sea (right) blue mussels, 1 and 4 h after exposure to different salinity (A, *n*=5; B, *n*=5) or temperature (C, *n*=6; D, *n*=5) treatments. Means±s.e.m.; data points represent individual biological replicates. Different capital letters represent significant differences between 1 h and 4 h time points (*P*<0.05). Different lowercase letters indicate significant differences among the five temperature/salinity treatment groups within the same time point (*P*<0.05). Statistical tests are detailed in Fig. 1.

Sperm VAP in the Baltic Sea population decreased at salinity 5 at both time points (1 h and 4 h), but remained relatively stable at salinities 9–21, with a modest but statistically significant decline observed between 1 h and 4 h of exposure (Fig. 3A). In North Sea mussels, VAP of the sperm decreased only at salinity 9 after 4 h, while remaining stable across all other salinities after 1 h and across salinities 17–35 after 4 h (Fig. 3B).

In both Baltic Sea and North Sea mussels, sperm VAP showed no significant effect of temperature after 1 h of exposure. In Baltic Sea mussel sperm, VAP decreased after 4 h of exposure to 25°C compared with 5–20°C (Fig. 3C). In North Sea mussels, no effect of temperature on sperm VAP was found after 4 h (Fig. 3D). No significant interaction was observed between exposure time and salinity/temperature on sperm VAP (Table S5).

**Fig. 4.**
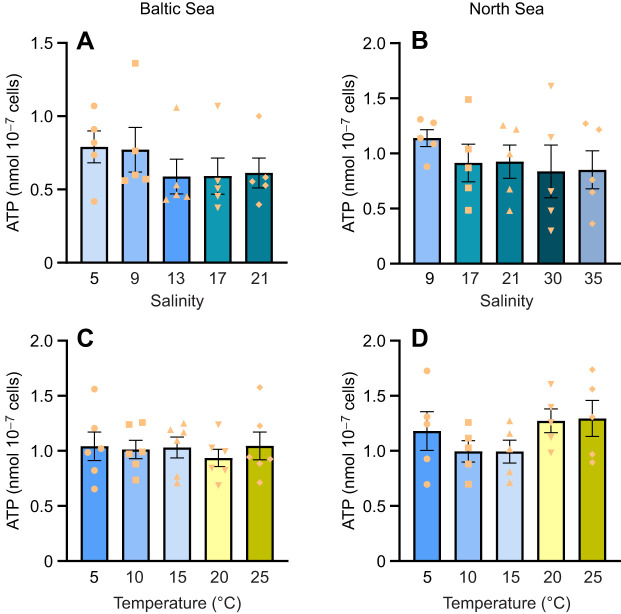
**ATP content of sperm exposed to different temperature or salinity conditions, for Baltic Sea and North Sea *M. edulis* populations.** ATP content of sperm from Baltic Sea (left) and North Sea (right) blue mussels, 3 h after exposure to different salinity (A, *n*=5; B, *n*=5) or temperature (C, *n*=6; D, *n*=5) treatments. Means±s.e.m.; data points represent individual biological replicates. Statistical tests are detailed in Fig. 1.

No significant difference was observed in the ATP content of sperm across different salinity or temperature treatments for both the Baltic Sea and North Sea populations (Fig. 4).

#### Fertilization success and embryonic development

**Fig. 5.**
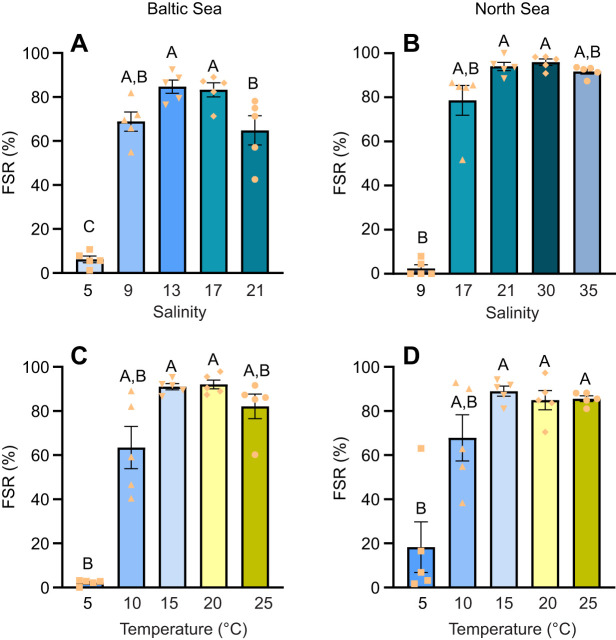
**Fertilization success rate (FSR) under different temperature or salinity conditions, for Baltic Sea and North Sea *M. edulis* populations.** FSR at 2h post-fertilization of blue mussels from the Baltic Sea (left) and the North Sea (right), after exposure to different salinity (A,B) or temperature (C,D) treatments (*n*=5). Means±s.e.m.; data points represent individual biological replicates. Different capital letters indicate significant differences among the five temperature/salinity treatment groups (*P*<0.05). Statistical tests are detailed in Fig. 1.

The optimal salinity range for fertilization in the Baltic Sea mussel population spanned from salinity 13 to salinity 17, with a FSR of 84.7±3.0% and 83.3±3.2%, respectively. Outside this optimal range, FSR decreased as seawater salinity increased or decreased. Notably, exposure to salinity 21 and salinity 5 resulted in a sharp decline in FSR, with values of 64.8±6.6% and 6.2±1.6%, respectively (Fig. 5A). In contrast, the optimal salinity range for fertilization in the North Sea mussel population was between salinity 21 and 30, yielding FSR values of 94.0±1.8% and 95.9±1.4%, respectively. These values were significantly higher than the FSR observed at salinity 9 (2.5±1.6%) (Fig. 5B). This contrasts sharply with the FSR in the Baltic Sea population, where FSR was 68.8±4.4% at salinity 9 (Fig. 5A).

Fertilization in both mussel populations showed resilience to elevated temperatures but was notably reduced in cold seawater conditions. FSR remained above 80% across the temperature range 15–25°C, with a sharp decline to 24–30% at 10°C (Fig. 5C,D). The lowest FSR, falling below 10% in the Baltic Sea population and below 20% in the North Sea population, was observed at 5°C (Fig. 5C,D).

The fertilization success in the temperature experiments for both mussel populations was not correlated with sperm motility traits, including the percentage of motile sperm, VCL, VAP and ATP content. However, in the salinity experiments for both populations, fertilization success was significantly correlated with sperm motility and VCL (also including VAP for the Baltic Sea population; *P*<0.05 for all), although no correlation was observed with ATP content (Table 1).

**Table 1.** Correlation between sperm performance traits and fertilization success rate under different salinity and temperature conditions for *Mytilus edulis* from the Baltic Sea and the North Sea

	Motility	VCL	VAP	Fertilization
**Baltic Sea**				
Salinity exposure	*N*=25	*N*=25	*N*=25	*N*=25
VCL	0.850**			
VAP	0.729**	0.759**		
Fertilization success	0.845**	0.832**	0.531**	
ATP	−0.322	−0.423*	−0.119	−0.359
Temperature exposure	*N*=30	*N*=30	*N*=30	*N*=25
VCL	0.888**			
VAP	0.786**	0.878**		
Fertilization success	0.278	0.188	0.082	
ATP	−0.354	−0.335	−0.079	−0.148
**North Sea**				
Salinity exposure	*N*=25	*N*=25	*N*=25	*N*=25
VCL	0.873**			
VAP	0.669**	0.860**		
Fertilization success	0.700**	0.479*	0.308	
ATP	−0.363	−0.392	−0.149	−0.261
Temperature exposure	*N*=25	*N*=25	*N*=25	*N*=25
VCL	0.853**			
VAP	0.643**	0.459*		
Fertilization success	−0.207	−0.146	−0.204	
ATP	−0.661**	−0.531**	−0.518**	−0.051

VCL, velocity curvilinear; VAP, velocity average path. *N* values are the sample size used to calculate the correlation coefficients. Numbers represent the correlation coefficient. Asterisks indicate significant correlations (*P<0.05 and **P<0.01; 2-tailed).

**Fig. 6.**
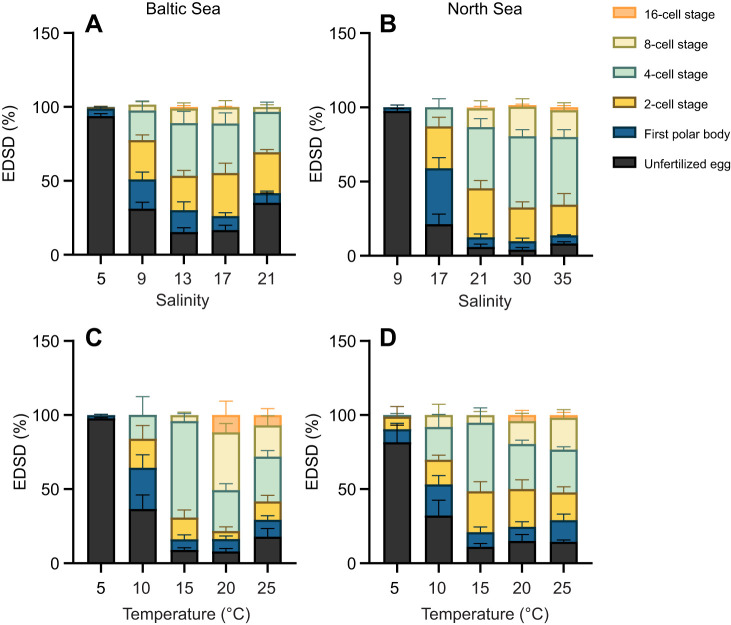
**Embryonic developmental stage distribution (EDSD) under different temperature or salinity conditions, for Baltic Sea and North Sea *M. edulis* populations.** EDSD at 2 h post-fertilization of blue mussels from the Baltic Sea (left) and North Sea (right), after exposure to different salinity (A,B) or temperature (C,D) treatments (*n*=5). Means±s.e.m.; data points represent individual biological replicates. Significant differences in outcomes are shown in Table S4.

Compared with salinity 5, where most eggs remained unfertilized, a high percentage of embryos reached the four-cell and eight-cell stage after 2 h at salinities 13–17 in the Baltic Sea mussel population, reaching 35.6±7.9% and 10.0±3.7% at salinity 13, and 33.5±7.1% and 10.9±4.5% at salinity 17 (Fig. 6A; Table S4). Embryonic development was slightly slower at salinities 9 and 21 compared with 13–17, though the differences were not statistically significant. In contrast, for the North Sea mussel population, the percentage of embryos in the four-cell and eight-cell stages was highest at salinities 30–35, significantly exceeding those at salinity 9 (Fig. 6B; Table S4).

Unlike the Baltic Sea population, where fertilization and embryonic development were stalled at 5°C, in the North Sea population, a small fraction of fertilized eggs successfully progressed to the two- and four-cell stages (8.5±6.9% and 1.0±1.0%, respectively) 2 h post-fertilization. A significant and abrupt increase in the percentage of eight-cell stage embryos was observed in the Baltic Sea mussel population as seawater temperature rose from 15°C to 20°C, increasing from 4.02±1.95% to 39.01±6.01%. Concurrently, the percentage of four-cell stage embryos decreased from 65.20±5.06% to 27.56±4.34%, indicating that more than 50% of the four-cell stage embryos successfully progressed to the eight-cell stage. Additionally, the percentage of eight-cell stage embryos in the 20°C and 25°C treatments was significantly higher than that in the 5°C and 10°C treatments (Fig. 6C; Table S4). Similarly, in the North Sea population, embryos displayed faster development under warmer seawater conditions. The percentage of eight-cell stage embryos in the 25°C treatment was significantly higher than that in the 5°C treatment (21.55±5.46% versus 0). The highest percentage of four-cell stage embryos was observed in the 15°C treatment, at 46.33±9.95%, compared with 1.03±1.03% in the 5°C treatment (Fig. 6D; Table S4). In both populations, embryos generally showed optimal development in warmer seawater temperatures (15–25°C).

### Combined effects of temperature and osmolarity on sperm mitochondrial function of mussels from the Baltic Sea

**Fig. 7.**
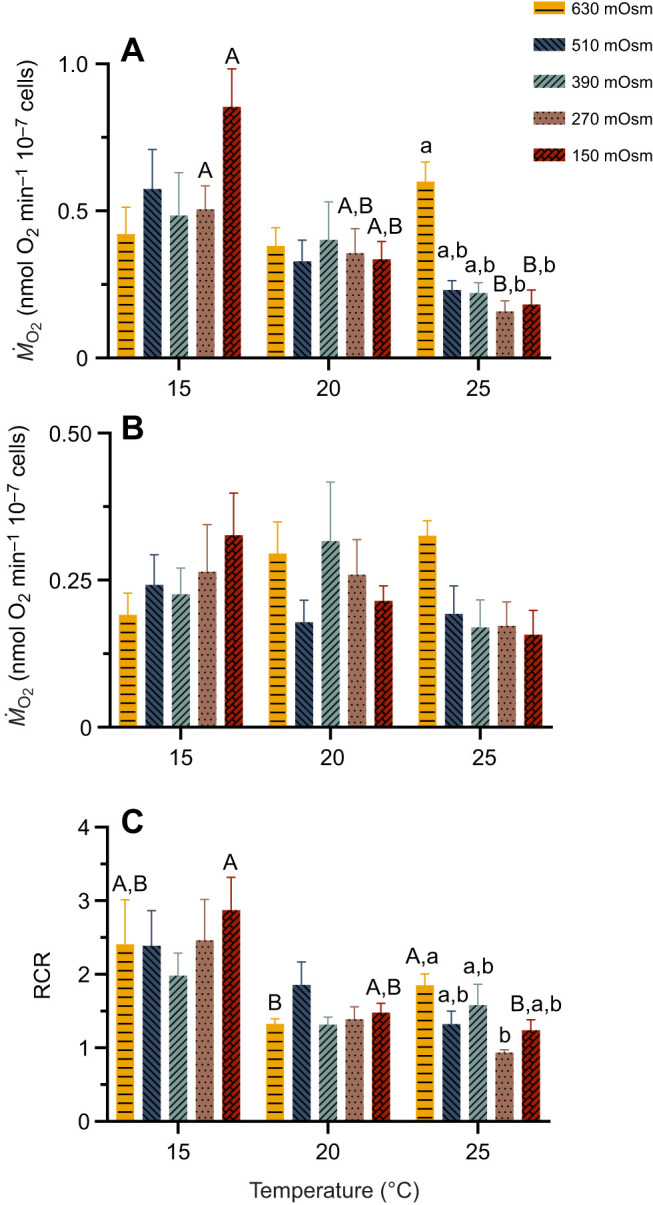
**Combined effects of temperature and osmolarity on sperm mitochondrial function of *M. edulis* from the Baltic Sea.** (A) Mitochondrial oxidative phosphorylation (OXPHOS) respiration, (B) LEAK I+II respiration and (C) respiratory control ratio (RCR) of sperm exposed to different temperature and osmolarity conditions. Means±s.e.m. (*n*=5–8). Different capital letters represent significant differences among the three temperature treatment groups at the same osmolarity (*P*<0.05). Different lowercase letters indicate significant differences among the five osmolarity treatment groups at the fixed temperature. Statistical tests are detailed in Fig. 1.

Sperm OXPHOS respiration was significantly reduced at 25°C when combined with low osmolarity (0.158±0.036 and 0.182±0.049 nmol O_2_ min^−1^ 10^−7^ cells at 270 mOsm and 150 mOsm, corresponding to salinity 9 and 5) relative to the values measured at 15°C (0.505±0.080 and 0.854±0.129 nmol O_2_ min^−1^ 10^−7^ cells at 270 mOsm and 150 mOsm, respectively). Notably, significant effects of osmolarity on sperm OXPHOS respiration were observed only at 25°C, where respiration in the 630 mOsm treatment was significantly higher than that in the 270 mOsm and 150 mOsm treatments (Fig. 7A). In contrast, no significant differences in LEAK state respiration was found in the Baltic Sea mussel sperm mitochondria across temperature and osmolarity treatments (Fig. 7B). The RCR of sperm mitochondria showed a general decline with increasing temperature (Fig. 7C). While osmolarity had minimal impact on RCR at 15°C and 20°C, it became a significant determinant at 25°C. The combined effects of elevated temperature (25°C) and low osmolarity (270 mOsm and 150 mOsm) produced RCR values close to 1, indicative of a loss of mitochondrial coupling (Fig. 7C).

**Fig. 8.**
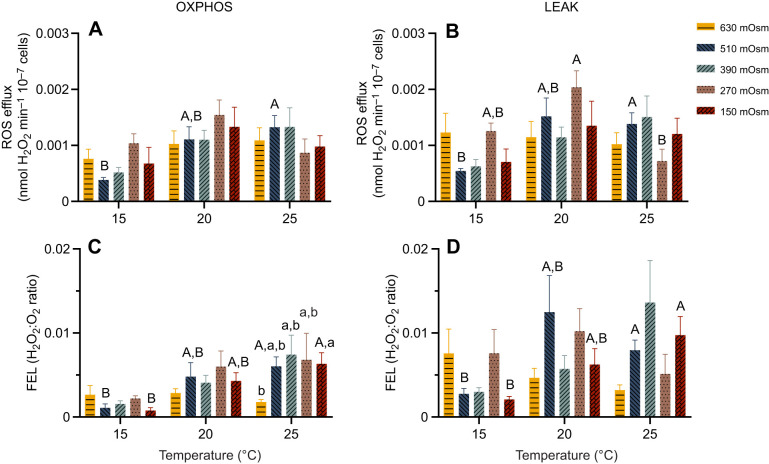
**Combined effects of temperature and osmolarity on sperm mitochondrial function of *M. edulis* from the Baltic Sea.** (A,B) Reactive oxygen species (ROS) efflux in sperm mitochondria in OXPHOS (A) and LEAK I+II (B) state under distinct temperature and osmolarity treatments. (C,D) Fractional electron leak (FEL; expressed as H_2_O_2_:O_2_ ratio) of sperm mitochondria in OXPHOS status (C) and LEAK I+II status (D) under distinct temperature and osmolarity treatments. Means±s.e.m. (*n*=5–8). Different capital letters represent significant differences among the three temperature treatment groups at the same osmolarity (*P*<0.05). Different lowercase letters indicate significant differences among the five osmolarity treatment groups at the fixed temperature (*P*<0.05). Statistical tests are detailed in Fig. 1.

The ROS efflux of sperm mitochondria in the OXPHOS state showed an increasing trend with rising temperature in higher osmolarity treatments (390–630 mOsm). In contrast, at lower osmolarities (150–270 mOsm), ROS efflux peaked at 20°C and showed a decreasing trend at 25°C, although the difference was not statistically significant (Fig. 8A). Similarly, in the OXPHOS state, FEL remained consistently low in the 630 mOsm treatment across all tested temperatures. A significant increase in FEL with warming was observed at 150 and 510 mOsm, whereas no significant temperature-dependent changes were found at 270 and 390 mOsm (Fig. 8C). In the LEAK state, ROS efflux remained stable in mitochondria exposed to 630 mOsm, and at this osmolarity level, FEL showed a decreasing trend with increasing temperature, although this change was not statistically significant (Fig. 8B,D). In the LEAK state, mitochondria exposed to 510 mOsm showed a significant increase in ROS efflux with warming (Fig. 8B), whereas at 270 mOsm, ROS efflux peaked at 20°C and declined significantly at 25°C. FEL in the LEAK state showed a similar pattern at lower osmolarities (150–510 mOsm), increasing with temperature and peaking at either 20°C (270 and 510 mOsm) or 25°C (150 and 390 mOsm).

Correlational analysis revealed that sperm mitochondrial respiration in the OXPHOS and LEAK states were strongly correlated (*P*<0.01), while RCR showed a significant correlation with mitochondrial respiration in the OXPHOS state but not the LEAK state. FEL in the OXPHOS state was positively correlated with ROS efflux and FEL LEAK, but negatively correlated with mitochondrial respiration and RCR. FEL in the LEAK state exhibited similar significant correlations with FEL OXPHOS, except for RCR. ROS efflux values in the OXPHOS and LEAK states were significantly correlated with each other but showed no significant correlations with other traits (Table 2).

**Table 2.** Correlations among all sperm mitochondrial traits under different osmolarity and temperature conditions for *M. edulis* from the Baltic Sea

	*Ṁ*_O_2__ LEAK	*Ṁ*_O_2__ OXPHOS	RCR	ROS LEAK	ROS OXPHOS	FEL LEAK	FEL OXPHOS
*Ṁ*_O_2__ LEAK	1						
*Ṁ*_O_2__ OXPHOS	0.804**	1					
RCR	0.028	0.574**	1				
ROS LEAK	0.026	0.01	−0.042	1			
ROS OXPHOS	0.14	0.041	−0.14	0.872**	1		
FEL LEAK	−0.636**	−0.525**	−0.021	0.710**	0.539**	1	
FEL OXPHOS	−0.500**	−0.699**	−0.482**	0.578**	0.634**	0.789**	1

*n*=92 for *Ṁ*_O_2__ LEAK, *Ṁ*_O_2__ OXPHOS and RCR; *n*=89 for ROS LEAK, ROS OXPHOS, FEL LEAK and FEL OXPHOS. Numbers represent the correlation coefficient. Asterisks indicate significant correlations (**P<0.01) (2-tailed); minus signs indicate a negative correlation.

## DISCUSSION

Measuring the performance of gametes under varying environmental conditions is crucial for understanding the reproductive success of marine invertebrates, particularly broadcast spawners such as mussels, whose gametes are directly exposed to seawater during external fertilization. Climate change introduces multiple stressors, such as temperature and salinity variation, which can significantly influence gamete performance. These factors are particularly relevant in light of gametes' sensitivity to environmental conditions (Boni et al., 2016; Cuccaro et al., 2022; Gallo et al., 2020; Tackett et al., 2024). Although previous research has highlighted the critical role of gamete quality in fertilization success (Budhwar et al., 2017; Gallo et al., 2020; Lewis and Ford, 2012), the specific effects of abiotic stressors on male fertility, including sperm metabolism and performance, remained underexplored. Our study demonstrates that temperature and salinity variation significantly influence the gamete performance of *M. edulis* populations from the North Sea and the Baltic Sea. Additionally, population-specific differences in tolerance to these environmental factors were observed, highlighting the adaptations of these populations to the differing salinity conditions in their respective habitats.

### Population-specific effects of salinity on sperm performance and fertilization

Our study demonstrates that adaptation to distinct salinity regimes in the maritime North Sea population and the brackish Baltic Sea population is reflected in differing optimal salinity ranges for sperm performance, fertilization success and embryonic and larval development in *M. edulis*. *Mytilus edulis* sperm from the brackish Baltic Sea population was characterized by a broad salinity window for optimal motility and velocity parameters (VCL and VAP), which remained stable between salinities of 9 and 21, with a significant decline observed at salinity 5. Despite this decline in motility, key metabolic parameters, including OXPHOS, resting LEAK respiration and mitochondrial coupling, remained stable across salinities 5–21 at a control temperature of 15°C. Additionally, ROS levels and fractional electron leak were unaffected, indicating that the decrease in sperm performance at salinity 5 is not attributable to disruptions in aerobic metabolism or oxidative stress. Furthermore, ATP content in Baltic Sea mussel sperm was stable across all salinities tested, suggesting that the observed decline in motility at salinity 5 is likely to be mediated by mechanisms unrelated to energy depletion. Similar results were observed in for Eastern oyster (*Crassostrea virginica*) sperm motility, where the low salinity of 10 significantly reduced sperm motility and progressive movement, while ATP levels remained unaffected (Tackett et al., 2024). This could be explained by the impairment of sperm motility signaling under low salinity, which is upstream of ATP-dependent sperm motility (Boulais et al., 2019). Additionally, low salinity inhibited sperm motility of *C. virginica* by disrupting ion homeostasis (K^+^, Ca^2+^, Na^+^) and altering osmotic pressure (Nichols et al., 2021), while Na^+^ and Ca^2+^ were involved in the regulation of [pH]_i_ and activation of axonemal proteins, respectively (Alavi et al., 2014; Boulais et al., 2018). These factors collectively impair sperm activation and reduce motility efficiency. Further investigation is needed to determine the possible mechanisms underlying the observed salinity-dependent variation in sperm performance unrelated to energy metabolism.

The fertilization success rate of the Baltic Sea *M. edulis* population also showed a relatively broad tolerance range between salinities 9 and 21, though a declining trend was observed at the extremes of this range. Embryonic development progressed most rapidly at salinities 13–17, while development at salinities 9 and 21 was notably slower. These findings suggest that the optimal salinity window for external fertilization and embryonic development in this brackish population is 13–17. In contrast, salinity 5 led to extremely low fertilization success, with embryonic development either stalling or being substantially delayed at the first polar body stage, identifying salinity 5 as a critical threshold at which reproduction is severely impaired. This threshold aligns with the critical salinity for marine macrozoobenthos initially proposed by Remane (see Telesh et al., 2011), which identifies a species diversity minimum within salinity range 5–8 due to physiological constraints. This highlights the challenges imposed by low salinity environments on the reproductive success of *M. edulis* populations in brackish ecosystems.

The sperm of the North Sea *M. edulis* population showed an optimal performance window shifted toward higher salinities (30–35) compared with the Baltic Sea population, with a gradual decline in sperm performance was observed between salinities 21 and 9. Among the measured parameters, sperm motility showed the strongest salinity-dependent variation, gradually declining below salinity 30 within 1 h of exposure. In contrast, declines in velocity parameters (VCL and VAP) were only observed after 4 h of exposure to salinities below 21. Fertilization success in the North Sea population was high within the salinity range 17–35, with modest reductions at the extremes (17 and 35) compared with the intermediate range (21–30). However, embryonic development at salinity 17 was delayed, indicating that reproductive success diminishes outside the optimal salinity range of 21–35. At salinity 9, fertilization success was severely disrupted, with only 2.48±1.62% of eggs fertilized, and development stalled at the first polar body stage. These results suggest that salinity 9 falls below the reproductive adaptation threshold for the North Sea population, highlighting significant differences in salinity tolerance and reproductive performance between the North Sea and Baltic Sea populations.

In both studied populations, salinity-dependent variation in fertilization success was strongly and positively correlated with sperm motility and velocity parameters (VCL and VAP). While sperm motility and velocity were also strongly correlated with each other, sperm motility emerged as the strongest predictor of fertilization success, explaining 85% of the variation in fertilization rates in the Baltic Sea population and 66% in the North Sea population. Notably, sperm ATP content showed no correlation with either sperm motility or fertilization success across different salinity exposures in both populations. This finding supports the hypothesis that salinity-induced disruptions in sperm motility, probably driven by osmotic or ionic stress, rather than energy deficiency, underlie the observed variation in sperm performance. These results also show that the salinity-induced limitations on the reproductive success of *M. edulis* are predominantly dependent on sperm motility and its associated performance metrics.

The pronounced differentiation in sperm and embryo salinity tolerance between the studied *M. edulis* populations is consistent with previous reports of genetic and phenotypic differentiation between the mussel populations from the Baltic and North Seas, encompassing variation in morphology, growth, metabolism and genetic composition (Johannesson et al., 1990; Kautsky et al., 1990; Koehn and Gaffney, 1984; Rodhouse et al., 1986; Varvio et al., 1988). However, these differences may not only reflect local adaptation but also genetic background differences, as the Baltic Sea population is part of a known hybrid zone (*M. edulis*×*M. trossulus*), with the mussels showing ∼70% of nuclear markers and 100% of mitochondrial markers of *M. edulis* (Stuckas et al., 2017), while the North Sea population is genetically pure *M. edulis* (Knöbel et al., 2020). Sperm motility emerged as highly sensitive to low salinities, with critical thresholds identified at salinity 5 for the Baltic Sea population and salinity 9 for the North Sea population, directly influencing fertilization success. Embryonic development of *M. edulis* was also salinity dependent, and integrating sperm performance and embryonic development parameters indicated optimal salinity ranges for reproduction of 13–17 in the Baltic Sea population and 21–35 in the North Sea population. The observed differences in optimal salinity range between the two populations may also reflect long-term local adaptation to distinct environmental salinity regimes. Baltic Sea mussels experience consistently low and fluctuating salinity, leading to enhanced osmoregulatory capacity and reproductive performance at lower salinities. The North Sea mussels are adapted to stable, high-salinity conditions and thus show reduced performance under hyposaline stress.

### Effects of temperature on sperm performance and fertilization

The temperature dependence of sperm performance revealed similar temperature tolerance for *M. edulis* sperm from the Baltic and North Sea populations, which may be due to the shared seasonal temperature fluctuations (5–15°C) they experience during their reproductive season. The selected experimental temperatures thus realistically represent both gradual seasonal warming and occasional heatwaves, which may exert comparable thermal selection pressures on the two populations. Sperm from both populations showed resilience to seawater temperature variation within the range 5–20°C, while sperm performance was significantly impaired at an elevated temperature of 25°C. The lowest tested temperature (5°C) is below the threshold for spawning induction in field populations of *M. edulis*. Natural spawning events typically occur in late spring, when seawater temperature reaches 9.5–12.5°C (Chipperfield, 1953). Nevertheless, our results indicate that at 5°C, the mussel sperm maintain activity levels comparable to those observed within the optimal spawning temperature range (10–15°C) for this species. The mechanisms underlying this cold tolerance remain unclear; however, it may serve as a protective trait to preserve sperm function during occasional cold spells in spring.

The delay in warming-induced sperm impairment may account for the absence of a significant effect of elevated temperature (25°C) on fertilization success when compared with the typical spawning temperature range (10–15°C) or moderate warming (20°C). Conversely, fertilization success at 2 h post-fertilization was markedly reduced at 5°C, despite high sperm motility and velocity under these conditions. This apparent mismatch between temperature effects on sperm activity and fertilization success is further reflected in the lack of a significant correlation between sperm motility and fertilization success, in contrast to the clear relationship observed in salinity variation treatments. These findings suggest that, in the context of temperature, the reduction in fertilization success is probably attributable to the disruption of fertilization-specific processes such as induction of the acrosomal reaction, sperm–egg binding and fusion or egg activation (Hirohashi et al., 2008), rather than a decline in sperm quality. Similar results were reported in the sea urchin *Anthocidaris crassispina*, where low temperatures (0–10°C) significantly reduced the number of sperm bound to the egg coat and the rate of the acrosome reaction (Mita et al., 1984). Additionally, it should be considered that in ectotherms such as mussels, low temperature can slow down fertilization and embryonic development. Thus, the apparent arrest of development observed at 5°C after the 2 h fertilization period could reflect a temperature-dependent decrease in cell division rate rather than complete inhibition. Nevertheless, it is worth noting that the Baltic Sea population was more sensitive to the cold-induced delay in the onset of cell division than the North Sea one.

Notably, embryonic development generally slows under low temperature conditions as a result of reduced metabolism and enzyme activity, which delays cell division. For instance, in the basket cockle, *Clinocardium nuttallii*, early embryogenesis was significantly delayed under low-temperature conditions, with cleavage stages reached much later than at optimal temperatures (Liu et al., 2008). Similarly, the sea urchin *Arbacia dufresnii* shows markedly delayed cleavage and development at low temperature (12°C versus 17°C) (Pía-Fernández et al., 2021). These findings align with our observations in *M. edulis* mussels and support the view that the apparent arrest of embryonic development at 5°C may reflect a pronounced temperature-dependent delay in early cleavage rather than a true developmental inhibition.

### Does mitochondrial impairment contribute to a salinity- and temperature-induced decrease in sperm quality?

Mitochondria play a key role in sperm activity, primarily through ATP generation via OXPHOS, which is strictly oxygen dependent (Kong and Sokolova, 2024). This link between mitochondrial aerobic respiration and ATP production is crucial for sperm function. The activity of key mitochondrial enzymes, such as citrate synthase and succinate dehydrogenase (Complex II), has been linked to sperm performance (Ruiz-Pesini et al., 1998, 2000). Furthermore, mitochondrial respiration, measured by oxygen consumption, and respiratory efficiency are strongly correlated with sperm motility (Stendardi et al., 2011). Inhibition of the electron transport system (ETS) with agents such as rotenone and oligomycin has been shown to negatively affect mussel sperm motility (Kong and Sokolova, 2024).

Environmental stressors such as temperature and osmotic changes can impair mitochondrial respiration, coupling efficiency and ATP synthesis in marine ectotherms (Bal et al., 2021; Ballantyne and Moyes, 1987; Heise et al., 2003; Ivanina et al., 2012; Pörtner et al., 1999; Sokolova, 2018), which may contribute to reduced sperm performance. Stress can also elevate ROS production, as electron leakage from the ETS generates ROS. Excessive ROS can lead to oxidative damage, such as lipid peroxidation, impairing sperm function (Aitken, 2017; Dutta et al., 2020; Fernández et al., 2024). Moreover, ROS production has been shown to negatively correlate with sperm morphology and motility (Aziz et al., 2004; Gallo et al., 2021). While sperm motility and mitochondrial metabolism have been studied in various marine invertebrates under stress (Binet et al., 2014; Gallo et al., 2018; Schlegel et al., 2015), the relationship between mitochondrial function and sperm motility remains underexplored. Our investigation of the combined effects of temperature and osmolarity on mitochondrial function in *M. edulis* sperm suggests that mitochondrial dysfunction alone does not fully account for the decline in sperm performance observed at elevated temperatures or reduced salinities. In Baltic Sea mussels, the decrease in sperm performance at low salinities (5) was not linked to disruption to mitochondrial metabolism. Baltic Sea mussel sperm mitochondria displayed high osmotic tolerance, maintaining stable OXPHOS rates and low ROS production across a wide range of salinities. While mitochondrial performance in North Sea mussel sperm under different salinities was not assessed, studies of somatic tissues in *M. edulis* indicate that mitochondria in North Sea mussels also exhibit broad osmotic tolerance (160–1200 mOsm) (Wiesenthal et al., 2025). Consequently, the observed decline in sperm motility at low salinities (5 in Baltic Sea and 9 in North Sea mussels) is probably due to the limited osmoregulatory capacity typical of marine osmoconformers, rather than oxidative stress or mitochondrial dysfunction.

Elevated temperature reduced mitochondrial ATP synthesis capacity in mussel sperm, as indicated by lower OXPHOS rates at 25°C compared with 15°C, along with a decline in coupling efficiency. However, this decline was only observed when warming was combined with reduced salinity (5–9). Despite this mitochondrial disruption, sperm ATP levels remained unaffected, suggesting that the observed decline in motility and velocity after 4 h at 25°C was not due to an energy deficit. This implies that mitochondrial capacity was sufficient to sustain ATP production even under suboptimal temperature conditions. At 25°C, mitochondrial impairment was further indicated by increased fractional electron leak at salinities of 5–17, which led to higher relative ROS production compared with O_2_ consumption. While excessive ROS production has been shown to cause oxidative damage in spermatozoa (e.g. lipid peroxidation), impairing sperm function (Aitken, 2017; Dutta et al., 2020; Fernández et al., 2024), our data did not provide direct evidence linking ROS efflux to impaired sperm motility. However, mitochondrial results showed that both sperm mitochondrial ROS efflux and fractional electron leak increased with temperature, suggesting that elevated temperature enhances electron leakage, potentially pushing it closer to the threshold for oxidative damage in sperm.

Overall, our findings demonstrate that the observed reductions in sperm motility, velocity and fertilization success of the blue mussels from the Baltic and North Sea under varying salinity or temperature conditions are not primarily driven by impaired mitochondrial function. Although high temperature decreased mitochondrial coupling efficiency and increased ROS production and FEL, these changes did not significantly affect ATP production. Thus, the mitochondrial tolerance appears sufficient for sperm energy demands, suggesting that other mechanisms may contribute to impaired reproduction under distinctive temperature and salinity conditions, especially for fertilization failure.

As fertilization is a complex process and requires well-coupled gametes, future studies should focus on exploring the mechanisms of fertilization failure under cold and low-salinity seawater environments, such as the potential loss of acrosomal enzyme activity required for successful fertilization, particularly under cold stress. Additionally, investigating how these factors interact with sperm–egg recognition and early embryonic development will be essential for understanding the full impact of environmental fluctuations on mussel reproduction.

## Supplementary Material

10.1242/jexbio.251452_sup1 Supplementary information

## References

[JEB251452C1] Aitken, R. J. (2017). Reactive oxygen species as mediators of sperm capacitation and pathological damage. *Mol. Reprod. Dev.* 84, 1039-1052. 10.1002/mrd.22871 28749007

[JEB251452C2] Alavi, S. M. H., Matsumura, N., Shiba, K., Itoh, N., Takahashi, K. G., Inaba, K. and Osada, M. (2014). Roles of extracellular ions and pH in 5-HT-induced sperm motility in marine bivalve. *Reproduction* 147, 331-345. 10.1530/REP-13-0418 24398874

[JEB251452C3] Aziz, N., Saleh, R. A., Sharma, R. K., Lewis-Jones, I., Esfandiari, N., Thomas, A. J., Jr and Agarwal, A. (2004). Novel association between sperm reactive oxygen species production, sperm morphological defects, and the sperm deformity index. *Fertil. Steril.* 81, 349-354. 10.1016/j.fertnstert.2003.06.026 14967372

[JEB251452C4] Bal, A., Panda, F., Pati, S. G., Das, K., Agrawal, P. K. and Paital, B. (2021). Modulation of physiological oxidative stress and antioxidant status by abiotic factors especially salinity in aquatic organisms. *Comp. Biochem. Physiol. C Toxicol. Pharmacol.* 241, 108971. 10.1016/j.cbpc.2020.108971 33421636

[JEB251452C5] Ballantyne, J. S. and Moyes, C. D. (1987). The effects of salinity acclimation on the osmotic properties of mitochondria from the gill of *Crassostrea virginica*. *J. Exp. Biol.* 133, 449-559. 10.1242/jeb.133.1.449

[JEB251452C6] Bechmann, R. K., Taban, I. C., Westerlund, S., Godal, B. F., Arnberg, M., Vingen, S., Ingvarsdottir, A. and Baussant, T. (2011). Effects of ocean acidification on early life stages of shrimp (*Pandalus borealis*) and mussel (*Mytilus edulis*). *J. Toxicol. Environ. Health A* 74, 424-438. 10.1080/15287394.2011.550460 21391089

[JEB251452C7] Binet, M. T. and Doyle, C. J. (2013). Effect of near-future seawater temperature rises on sea urchin sperm longevity. *Mar. Freshw. Res.* 64, 1-9. 10.1071/MF12121

[JEB251452C8] Binet, M. T., Doyle, C. J., Williamson, J. E. and Schlegel, P. (2014). Use of JC-1 to assess mitochondrial membrane potential in sea urchin sperm. *J. Exp. Mar. Biol. Ecol.* 452, 91-100. 10.1016/j.jembe.2013.12.008

[JEB251452C9] Boni, R., Gallo, A., Montanino, M., Macina, A. and Tosti, E. (2016). Dynamic changes in the sperm quality of *Mytilus galloprovincialis* under continuous thermal stress. *Mol. Reprod. Dev.* 83, 162-173. 10.1002/mrd.22604 26663619

[JEB251452C10] Boroda, A. V., Kipryushina, Y. O. and Odintsova, N. A. (2020). The effects of cold stress on *Mytilus* species in the natural environment. *Cell Stress Chaperones* 25, 821-832. 10.1007/s12192-020-01109-w 32297161 PMC7591686

[JEB251452C11] Boulais, M., Suquet, M., Arsenault-Pernet, E. J., Malo, F., Queau, I., Pignet, P., Ratiskol, D., Le Grand, J., Huber, M. and Cosson, J. (2018). pH controls spermatozoa motility in the Pacific oyster (*Crassostrea gigas*). *Biol. Open* 7, bio031427. 10.1242/bio.031427 29483075 PMC5898264

[JEB251452C12] Boulais, M., Demoy-Schneider, M., Alavi, S. M. H. and Cosson, J. (2019). Spermatozoa motility in bivalves: signaling, flagellar beating behavior, and energetics. *Theriogenology* 136, 15-27. 10.1016/j.theriogenology.2019.06.025 31234053

[JEB251452C13] Budhwar, S., Singh, V., Verma, P. and Singh, K. (2017). Fertilization failure and gamete health: is there a link. *Front. Biosci.* 9, 395-419. 10.2741/s494 28410126

[JEB251452C14] Cheng, G., Liang, Y., Zhang, H., Xu, C. and Li, Q. (2024). Effects of temperature and salinity on the larval early development, growth, and settlement of the diploid, triploid, and tetraploid Pacific oyster “Haida No. 2” strain. *Aquac. Int.* 32, 6097-6113. 10.1007/s10499-024-01457-9

[JEB251452C15] Chipperfield, P. N. J. (1953). Observations on the breeding and settlement of *Mytilus edulis* (L.) in British waters. *J. Mar. Biol. Assoc. U. K.* 32, 449-476. 10.1017/S002531540001465X

[JEB251452C16] Cuccaro, A., De Marchi, L., Oliva, M., Monni, G., Miragliotta, V., Fumagalli, G., Freitas, R. and Pretti, C. (2022). The influence of salinity on the toxicity of chemical UV-filters to sperms of the free-spawning mussel *Mytilus galloprovincialis* (Lamark, 1819). *Aquatic Toxicol.* 250, 106263. 10.1016/j.aquatox.2022.106263 35939883

[JEB251452C17] Dutta, S., Henkel, R., Sengupta, P. and Agarwal, A. (2020). Physiological role of ROS in sperm function. In *Male Infertility* (ed. S. Parekattil, S. Esteves, and A. Agarwal), pp. 337-345. Cham: Springer. 10.1007/978-3-030-32300-4_27

[JEB251452C18] Eads, A. R., Evans, J. P. and Kennington, W. J. (2016). Plasticity of fertilization rates under varying temperature in the broadcast spawning mussel, *Mytilus galloprovincialis*. *Ecol. Evol.* 6, 6578-6585. 10.1002/ece3.2375 27777731 PMC5058529

[JEB251452C19] Estabrook, R. W. (1967). [7] Mitochondrial respiratory control and the polarographic measurement of ADP: O ratios. *Methods Enzymol.* 10, 41-47. 10.1016/0076-6879(67)10010-4

[JEB251452C20] Fernández, I., Larrán, A. M., De Paz, P. and Riesco, M. F. (2024). The direct effects of climate change on tench (*Tinca tinca*) sperm quality under a real heatwave event scenario. *Animals* 14, 778. 10.3390/ani14050778 38473163 PMC10930877

[JEB251452C21] Frölicher, T. L., Fischer, E. M. and Gruber, N. (2018). Marine heatwaves under global warming. *Nature* 560, 360-364. 10.1038/s41586-018-0383-9 30111788

[JEB251452C22] Fusco, G. and Minelli, A. (2019). *The Biology of Reproduction*. Cambridge University Press.

[JEB251452C23] Gallo, A., Manfra, L., Boni, R., Rotini, A., Migliore, L. and Tosti, E. (2018). Cytotoxicity and genotoxicity of CuO nanoparticles in sea urchin spermatozoa through oxidative stress. *Environ. Int.* 118, 325-333. 10.1016/j.envint.2018.05.034 29960187

[JEB251452C24] Gallo, A., Boni, R. and Tosti, E. (2020). Gamete quality in a multistressor environment. *Environ. Int.* 138, 105627. 10.1016/j.envint.2020.105627 32151884

[JEB251452C25] Gallo, A., Esposito, M. C., Tosti, E. and Boni, R. (2021). Sperm motility, oxidative status, and mitochondrial activity: Exploring correlation in different species. *Antioxidants* 10, 1131. 10.3390/antiox10071131 34356364 PMC8301117

[JEB251452C26] Gregory, K. M., McFarland, K. and Hare, M. P. (2023). Reproductive phenology of the eastern oyster, *Crassostrea virginica* (Gmelin, 1791), along a temperate estuarine salinity gradient. *Estuaries Coasts* 46, 707-722. 10.1007/s12237-022-01163-w

[JEB251452C27] Heise, K., Puntarulo, S., Pörtner, H. O. and Abele, D. (2003). Production of reactive oxygen species by isolated mitochondria of the Antarctic bivalve *Laternula elliptica* (King and Broderip) under heat stress. *Comp. Biochem. Physiol. C Toxicol. Pharmacol.* 134, 79-90. 10.1016/S1532-0456(02)00212-0 12524020

[JEB251452C28] Hirohashi, N., Kamei, N., Kubo, H., Sawada, H., Matsumoto, M. and Hoshi, M. (2008). Egg and sperm recognition systems during fertilization. *Dev. Growth Differ.* 50, S221-S238. 10.1111/j.1440-169X.2008.01017.x 18494705

[JEB251452C29] Hobday, A. J., Alexander, L. V., Perkins, S. E., Smale, D. A., Straub, S. C., Oliver, E. C. J., Benthuysen, J. A., Burrows, M. T., Donat, M. G., Feng, M. et al. (2016). A hierarchical approach to defining marine heatwaves. *Prog. Oceanogr.* 141, 227-238. 10.1016/j.pocean.2015.12.014

[JEB251452C30] Ivanina, A. V., Kurochkin, I. O., Leamy, L. and Sokolova, I. M. (2012). Effects of temperature and cadmium exposure on the mitochondria of oysters (*Crassostrea virginica*) exposed to hypoxia and subsequent reoxygenation. *J. Exp. Biol.* 215, 3142-3154. 10.1242/jeb.071357 22660786

[JEB251452C31] Jastroch, M., Divakaruni, A. S., Mookerjee, S., Treberg, J. R. and Brand, M. D. (2010). Mitochondrial proton and electron leaks. *Essays Biochem.* 47, 53-67. 10.1042/bse0470053 20533900 PMC3122475

[JEB251452C32] Johannesson, K., Kautsky, N. and Tedengren, M. (1990). Genotypic and phenotypic differences between Baltic and North Sea populations of *Mytilus edulis* evaluated through reciprocal transplantations. II. Genetic variation. *Mar. Ecol. Prog. Ser.* 59, 211-219. 10.3354/meps059211

[JEB251452C33] Kautsky, N. (1982). Quantitative studies on gonad cycle, fecundity, reproductive output and recruitment in a Baltic *Mytilus edulis* population. *Mar. Biol.* 68, 143-160. 10.1007/BF00397601

[JEB251452C34] Kautsky, N., Johannesson, K. and Tedengren, M. (1990). Genotypic and phenotypic differences between Baltic and North Sea populations of *Mytilus edulis* evaluated through reciprocal transplantations. I. Growth and morphology. *Mar. Ecol. Prog. Ser.* 59, 203-210. 10.3354/meps059203

[JEB251452C35] Knöbel, L., Breusing, C., Bayer, T., Sharma, V., Hiller, M., Melzner, F. and Stuckas, H. (2020). Comparative de novo assembly and annotation of mantle tissue transcriptomes from the *Mytilus edulis* species complex (*M. edulis*, *M. galloprovincialis*, *M. trossulus*). *Mar. Genomics* 51, 100700. 10.1016/j.margen.2019.100700

[JEB251452C36] Koehn, R. K. and Gaffney, P. M. (1984). Genetic heterozygosity and growth rate in *Mytilus edulis*. *Mar. Biol.* 82, 1-7. 10.1007/BF00392757

[JEB251452C37] Kong, H. and Sokolova, I. M. (2024). Oxidative phosphorylation rather than glycolysis is the primary energy source for sperm motility in the mussels *Mytilus edulis*. *Comp. Biochem. Physiol. B Biochem. Mol. Biol.* 270, 110909. 10.1016/j.cbpb.2023.110909 37898360

[JEB251452C38] Kong, H. and Sokolova, I. M. (2025). Effects of hypoxia and anoxia on the reproductive performance of the blue mussel *Mytilus edulis*. *Sci. Total Environ.* 972, 179103. 10.1016/j.scitotenv.2025.179103 40088790

[JEB251452C39] Larsson, J., Lind, E. E., Corell, H., Grahn, M., Smolarz, K. and Lönn, M. (2017). Regional genetic differentiation in the blue mussel from the Baltic Sea area. *Estuarine Coast. Shelf Sci.* 195, 98-109. 10.1016/j.ecss.2016.06.016

[JEB251452C40] Lehmann, A., Myrberg, K., Post, P., Chubarenko, I., Dailidiene, I., Hinrichsen, H.-H., Hüssy, K., Liblik, T., Meier, H. E. M., Lips, U. et al. (2022). Salinity dynamics of the Baltic Sea. *Earth Syst. Dyn.* 13, 373-392. 10.5194/esd-13-373-2022

[JEB251452C41] Leite, C., Russo, T., Cuccaro, A., Pinto, J., Polese, G., Soares, A. M. V. M., Pretti, C., Pereira, E. and Freitas, R. (2024). The role of warming in modulating neodymium effects on adults and sperm of *Mytilus galloprovincialis*. *J. Environ. Manag.* 358, 120854. 10.1016/j.jenvman.2024.120854 38640759

[JEB251452C42] Lewis, C. and Ford, A. T. (2012). Infertility in male aquatic invertebrates: a review. *Aquat. Toxicol.* 120-121, 79-89. 10.1016/j.aquatox.2012.05.002 22640873

[JEB251452C43] Liu, W., Alabi, A. O. and Pearce, C. M. (2008). Fertilization and embryonic development in the basket cockle, *Clinocardium nuttallii*. *J. Shellfish Res.* 27, 393-397. 10.2983/0730-8000(2008)27[393:FAEDIT]2.0.CO;2

[JEB251452C44] Lymbery, R. A., Kennington, W. J. and Evans, J. P. (2021). The thermal environment of sperm affects offspring success: a test of the anticipatory paternal effects hypothesis in the blue mussel. *Biol. Lett.* 17, 20210213. 10.1098/rsbl.2021.0213 34228940 PMC8260270

[JEB251452C45] Meier, H. E. M., Kniebusch, M., Dieterich, C., Gröger, M., Zorita, E., Elmgren, R., Myrberg, K., Ahola, M. P., Bartosova, A., Bonsdorff, E. et al. (2022). Climate change in the Baltic Sea region: a summary. *Earth Syst. Dyn.* 13, 457-593. 10.5194/esd-13-457-2022

[JEB251452C46] Mita, M., Hino, A. and Yasumasu, I. (1984). Effect of temperature on interaction between eggs and spermatozoa of sea urchin. *Biol. Bull.* 166, 68-77. 10.2307/1541431

[JEB251452C47] Mooney, P. (2016). Fertilization success and gamete viability of the Pacific oyster (*Crassostrea gigas*) across a salinity gradient. *Degree thesis*, Novia University of Applied Sciences. https://urn.fi/URN:NBN:fi:amk-201604244899.

[JEB251452C48] Naumann, M., Umlauf, L., Mohrholz, V., Kuss, J., Siegel, H., Waniek, J. J. and Schulz-Bull, D. E. (2018). Hydrographic-hydrochemical assessment of the Baltic Sea 2017. *Meereswissenschaftliche Berichte* 107.

[JEB251452C49] Nichols, Z. G., Rikard, S., Alavi, S. M. H., Walton, W. C. and Butts, I. A. E. (2021). Regulation of sperm motility in Eastern oyster (*Crassostrea virginica*) spawning naturally in seawater with low salinity. *PLoS ONE* 16, e0243569. 10.1371/journal.pone.0243569 33735238 PMC7971463

[JEB251452C50] Ouillon, N., Sokolov, E. P., Otto, S., Rehder, G. and Sokolova, I. M. (2021). Effects of variable oxygen regimes on mitochondrial bioenergetics and reactive oxygen species production in a marine bivalve, *Mya arenaria*. *J. Exp. Biol.* 224, jeb237156. 10.1242/jeb.237156 33436367

[JEB251452C51] Parker, L. M., Ross, P. M. and O'Connor, W. A. (2009). The effect of ocean acidification and temperature on the fertilization and embryonic development of the Sydney rock oyster *Saccostrea glomerata* (Gould 1850). *Glob. Change Biol.* 15, 2123-2136. 10.1111/j.1365-2486.2009.01895.x

[JEB251452C52] Pechenik, J. A., Eyster, L. S., Widdows, J. and Bayne, B. L. (1990). The influence of food concentration and temperature on growth and morphological differentiation of blue mussel *Mytilus edulis* L. larvae. *J. Exp. Mar. Biol. Ecol.* 136, 47-64. 10.1016/0022-0981(90)90099-X

[JEB251452C53] Pía-Fernández, J., Belén-Chaar, F., Epherra, L., González-Aravena, J.-M. and Rubilar, T. (2021). Embryonic and larval development is conditioned by water temperature and maternal origin of eggs in the sea urchin *Arbacia dufresnii* (Echinodermata: Echinoidea). *Rev. Biol. Tropical* 69, 452-463. 10.15517/rbt.v69iSuppl.1.46384

[JEB251452C54] Pörtner, H. O., Hardewig, I. and Peck, L. S. (1999). Mitochondrial function and critical temperature in the Antarctic bivalve, *Laternula elliptica*. *Comp. Biochem. Physiol.* 124A, 179-189. 10.1016/S1095-6433(99)00105-1 12024288

[JEB251452C55] Ramesh, K., Hu, M. Y., Thomsen, J., Bleich, M. and Melzner, F. (2017). Mussel larvae modify calcifying fluid carbonate chemistry to promote calcification. *Nat. Commun.* 8, 1709. 10.1038/s41467-017-01806-8 29167466 PMC5700083

[JEB251452C56] Rick, J. J., Scharfe, M., Romanova, T., van Beusekom, J. E. E., Asmus, R., Asmus, H., Mielck, F., Kamp, A., Sieger, R. and Wiltshire, K. H. (2022). An evaluation of long-term physical and hydrochemical measurements at the Sylt Roads Marine Observatory (1973–2019), Wadden Sea, North Sea. *Earth Syst. Sci. Data Discussions* 15, 1037-1057. 10.5194/essd-2020-263

[JEB251452C57] Rodhouse, P. G., McDonald, J. H., Newell, R. I. E. and Koehn, R. K. (1986). Gamete production, somatic growth and multiple-locus enzyme heterozygosity in *Mytilus edulis*. *Mar. Biol.* 90, 209-214. 10.1007/BF00569129

[JEB251452C58] Ruiz-Pesini, E., Diez, C., Lapeña, A. C., Pérez-Martos, A., Montoya, J., Alvarez, E., Arenas, J. and López-Pérez, M. J. (1998). Correlation of sperm motility with mitochondrial enzymatic activities. *Clin. Chem.* 44, 1616-1620. 10.1093/clinchem/44.8.1616 9702947

[JEB251452C59] Ruiz-Pesini, E., Lapeña, A. C., Díez, C., Álvarez, E., Enríquez, J. A. and López-Pérez, M. J. (2000). Seminal quality correlates with mitochondrial functionality. *Clin. Chim. Acta* 300, 97-105. 10.1016/S0009-8981(00)00305-3 10958866

[JEB251452C60] Rutgersson, A., Kjellström, E., Haapala, J., Stendel, M., Danilovich, I., Drews, M., Jylhä, K., Kujala, P., Larsén, X. G., Halsnæs, K. et al. (2022). Natural hazards and extreme events in the Baltic Sea region. *Earth Syst. Dyn.* 13, 251-301. 10.5194/esd-13-251-2022

[JEB251452C61] Santos, I. A., Castellano, G. C. and Freire, C. A. (2013). Direct relationship between osmotic and ionic conforming behavior and tissue water regulatory capacity in echinoids. *Comp. Biochem. Physiol. A Mol. Integr. Physiol.* 164, 466-476. 10.1016/j.cbpa.2012.12.010 23261991

[JEB251452C62] Schlegel, P., Binet, M. T., Havenhand, J. N., Doyle, C. J. and Williamson, J. E. (2015). Ocean acidification impacts on sperm mitochondrial membrane potential bring sperm swimming behaviour near its tipping point. *J. Exp. Biol.* 218, 1084-1090. 10.1242/jeb.114900 25833135

[JEB251452C63] Seuront, L., Nicastro, K. R., Zardi, G. I. and Goberville, E. (2019). Decreased thermal tolerance under recurrent heat stress conditions explains summer mass mortality of the blue mussel *Mytilus edulis*. *Sci. Rep.* 9, 17498. 10.1038/s41598-019-53580-w 31767954 PMC6877631

[JEB251452C64] Shi, W., Han, Y., Guo, C., Zhao, X., Liu, S., Su, W., Wang, Y., Zha, S., Chai, X. and Liu, G. (2017). Ocean acidification hampers sperm-egg collisions, gamete fusion, and generation of Ca^2+^ oscillations of a broadcast spawning bivalve, *Tegillarca granosa*. *Mar. Environ. Res.* 130, 106-112. 10.1016/j.marenvres.2017.07.016 28750793

[JEB251452C65] Sokolova, I. (2018). Mitochondrial Adaptations to Variable environments and their role in animals’ stress tolerance. *Integr. Comp. Biol.* 58, 519-531. 10.1093/icb/icy017 29701785

[JEB251452C66] Sokolova, I. M. (2023). Ectotherm mitochondrial economy and responses to global warming. *Acta Physiol.* 237, e13950. 10.1111/apha.13950 36790303

[JEB251452C67] Sorte, C. J. B., Bernatchez, G., Mislan, K. A. S., Pandori, L. L. M., Silbiger, N. J. and Wallingford, P. D. (2019). Thermal tolerance limits as indicators of current and future intertidal zonation patterns in a diverse mussel guild. *Mar. Biol.* 166, 1-13. 10.1007/s00227-018-3452-6

[JEB251452C68] Sprung, M. (1983). Reproduction and fecundity of the mussel *Mytilus edulis* at Helgoland (North Sea). *Helgoländer Meeresuntersuchungen* 36, 243-255. 10.1007/BF01983629

[JEB251452C69] Sprung, M. and Bayne, B. L. (1984). Some practical aspects of fertilizing the eggs of the mussel *Mytilus edulis* L. *ICES J. Mar. Sci.* 41, 125-128. 10.1093/icesjms/41.2.125

[JEB251452C70] Steffen, J. B. M., Sokolov, E. P., Bock, C. and Sokolova, I. M. (2023). Combined effects of salinity and intermittent hypoxia on mitochondrial capacity and reactive oxygen species efflux in the Pacific oyster, *Crassostrea gigas*. *J. Exp. Biol.* 226, jeb246164. 10.1242/jeb.246164 37470191 PMC10445735

[JEB251452C71] Stendardi, A., Focarelli, R., Piomboni, P., Palumberi, D., Serafini, F., Ferramosca, A. and Zara, V. (2011). Evaluation of mitochondrial respiratory efficiency during in vitro capacitation of human spermatozoa. *Int. J. Androl.* 34, 247-255. 10.1111/j.1365-2605.2010.01078.x 20546047

[JEB251452C72] Stuckas, H., Knöbel, L., Schade, H., Breusing, C., Hinrichsen, H. H., Bartel, M., Langguth, K. and Melzner, F. (2017). Combining hydrodynamic modelling with genetics: can passive larval drift shape the genetic structure of Baltic *Mytilus* populations? *Mol. Ecol.* 26, 2765-2782. 10.1111/mec.14075 28238204

[JEB251452C73] Tackett, V. M. K., Montague, H. R., Stoeckel, J. A., Rikard, F. S., Tarnecki, A. M. and Butts, I. A. E. (2024). Salinity impacts gamete quality in eastern oyster, *Crassostrea virginica*. *Aquaculture* 589, 740869. 10.1016/j.aquaculture.2024.740869

[JEB251452C74] Telesh, I. V., Schubert, H. and Skarlato, S. O. (2011). Revisiting Remane's concept: evidence for high plankton diversity and a protistan species maximum in the horohalinicum of the Baltic Sea. *Mar. Ecol. Prog. Ser.* 421, 1-11. 10.3354/meps08928

[JEB251452C75] Thurman, C. L., Faria, S. C. and McNamara, J. C. (2013). The distribution of fiddler crabs (*Uca*) along the coast of Brazil: implications for biogeography of the western Atlantic Ocean. *Mar. Biodiversity Records* 6, e1. 10.1017/S1755267212000942

[JEB251452C76] Tidau, S., Brough, F. T., Gimenez, L., Jenkins, S. R. and Davies, T. W. (2023). Impacts of artificial light at night on the early life history of two ecosystem engineers. *Philos. Trans. R. Soc. B* 378, 20220363. 10.1098/rstb.2022.0363 PMC10613533 37899009

[JEB251452C77] Varvio, S.-L., Koehn, R. K. and Väinölä, R. (1988). Evolutionary genetics of the *Mytilus edulis* complex in the North Atlantic region. *Mar. Biol.* 98, 51-60. 10.1007/BF00392658

[JEB251452C78] Ventura, A., Schulz, S. and Dupont, S. (2016). Maintained larval growth in mussel larvae exposed to acidified under-saturated seawater. *Sci. Rep.* 6, 23728. 10.1038/srep23728 27020613 PMC4810423

[JEB251452C79] Westerbom, M., Kilpi, M. and Mustonen, O. (2002). Blue mussels, *Mytilus edulis*, at the edge of the range: population structure, growth and biomass along a salinity gradient in the north-eastern Baltic Sea. *Mar. Biol.* 140, 991-999. 10.1007/s00227-001-0765-6

[JEB251452C80] Wiesenthal, A. A., Timm, S. and Sokolova, I. M. (2025). Osmotolerance reflected in mitochondrial respiration of *Mytilus* populations from three different habitat salinities. *Mar. Environ. Res.* 205, 106968. 10.1016/j.marenvres.2025.106968 39883997

[JEB251452C81] Wilson-Leedy, J. G. and Ingermann, R. L. (2007). Development of a novel CASA system based on open source software for characterization of zebrafish sperm motility parameters. *Theriogenology* 67, 661-672. 10.1016/j.theriogenology.2006.10.003 17137620

