# Supplementary material for: Variation in sperm performance and mitochondrial metabolism of Mytilus spp. from the North and Baltic Seas under different environmental scenarios
Source: J Exp Biol. 2026 Feb 10;229(3):jeb251452. doi: 10.1242/jeb.251452 (PMC12951606; doi:10.1242/jeb.251452)
Supplement: Supplementary information [file jexbio-229-251452-s1.pdf]

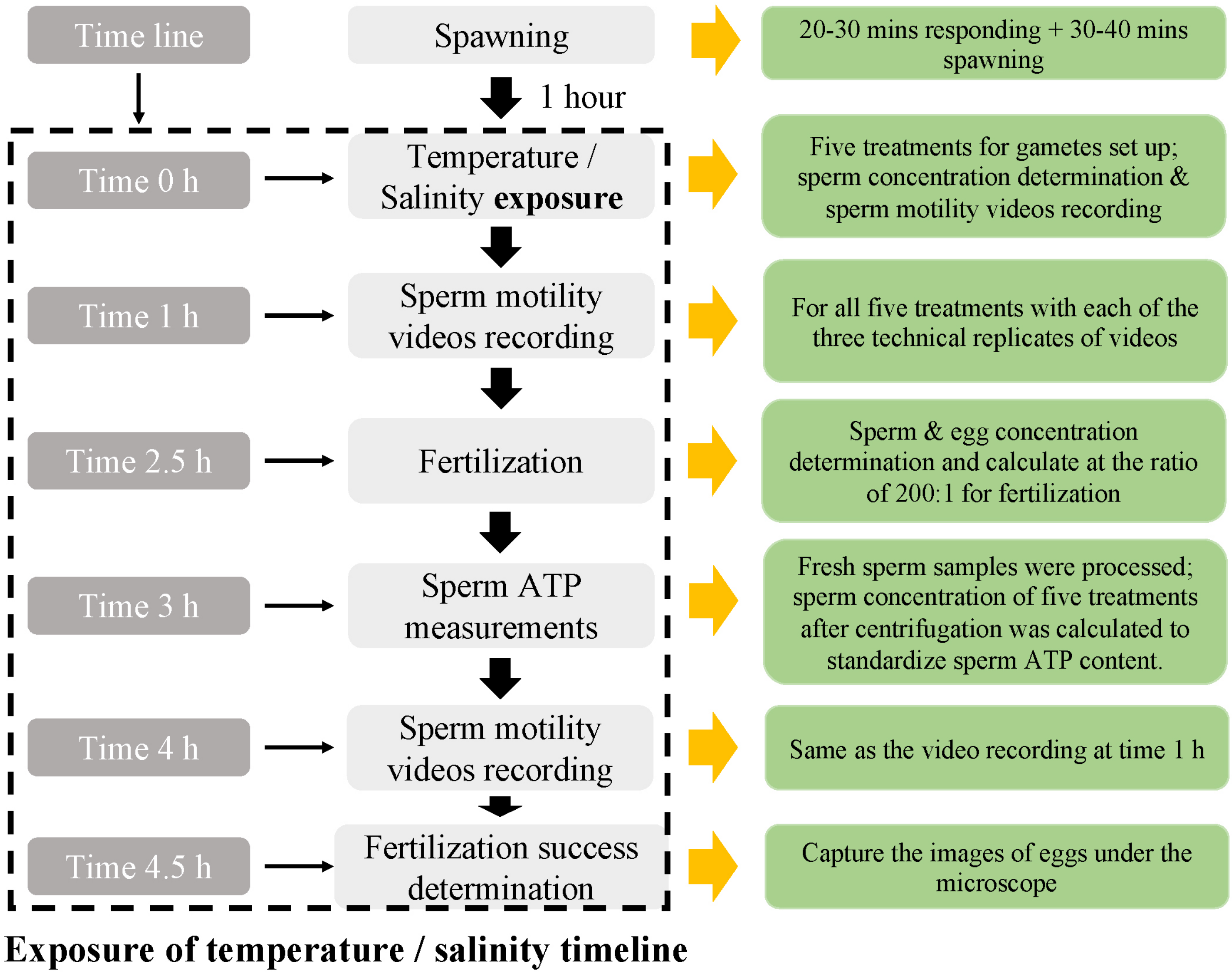

Fig. S1. Experimental timeline for sperm performance and fertilization assays. Adult mussels were induced to spawn by the thermal shock method over a period of 1 h (including ~20–30 minutes of thermal response and ~30–40 minutes of gamete release). Collected sperm and eggs were then divided into five treatments for temperature or salinity exposure. Sperm motility was recorded at 1 h and 4 h post-exposure, fertilization was performed at time 2.5 h, sperm ATP content was measured at time 3 h, and fertilization success was assessed at time 4.5 h.

Journal of Experimental Biology • Supplementary information

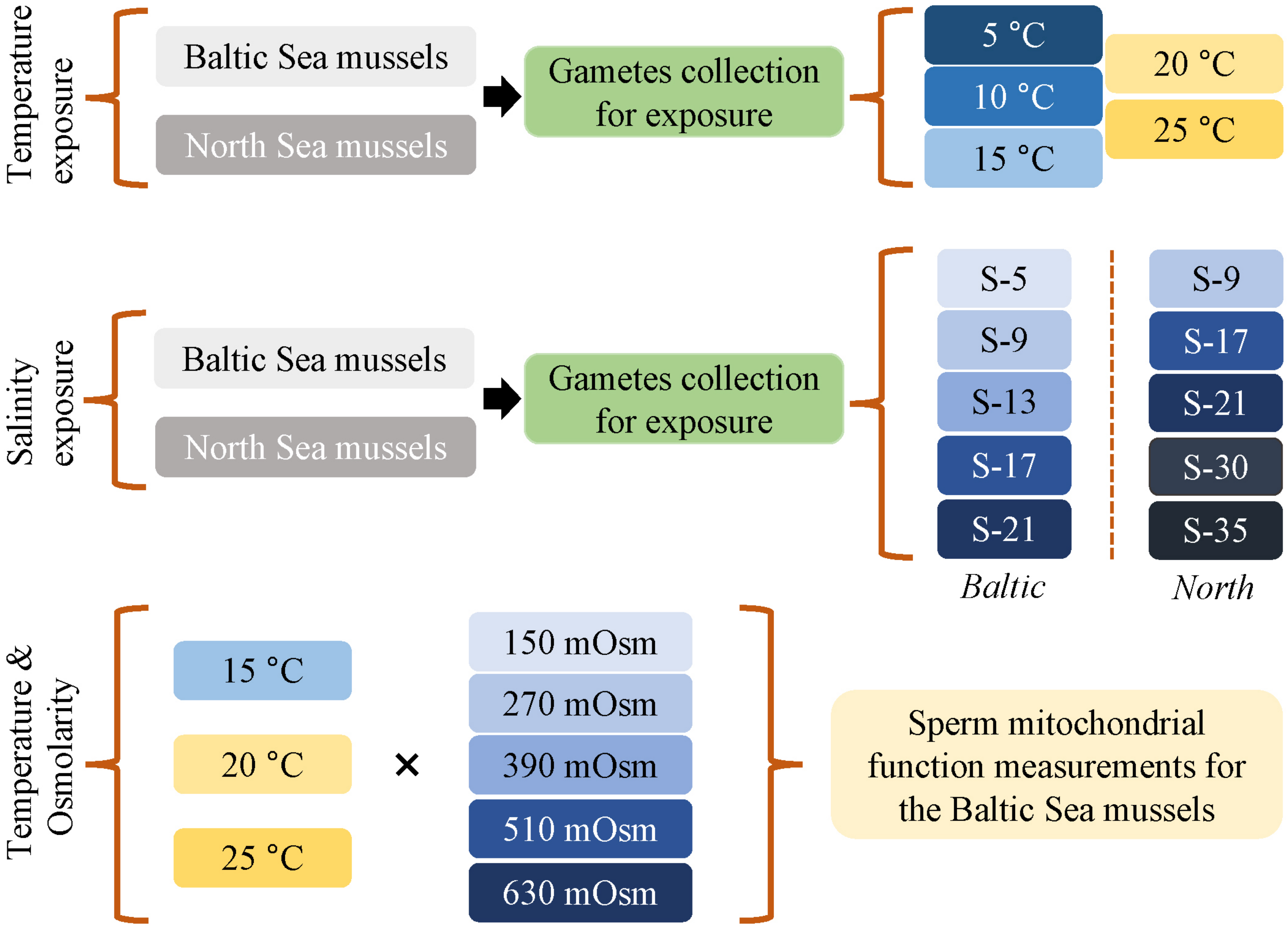

Fig. S2. The effects of salinity/osmolarity and temperature on sperm performance and mitochondrial metabolism in blue mussel *M. edulis* from the North Sea and the Baltic Sea populations.

Journal of Experimental Biology • Supplementary information

Table S1. Temperature and salinity conditions in experimental exposures of gamete suspensions (Gametes) and fertilization media (Fertilization) of *M. edulis* from the Baltic Sea (BS) and the North Sea (NS) population. Salinities are in practical salinity units, n - the number of biological replicates.

BS: Salinity	Gametes	n	Fertilization	n
5	5.11 ± 0.02	5	5.12 ± 0.02	5
9	9.03 ± 0.05	5	9.10 ± 0.01	5
13	12.93 ± 0.02	5	13.12 ± 0.02	5
17	16.98 ± 0.03	5	17.03 ± 0.05	5
21	20.87 ± 0.02	5	20.91 ± 0.02	5
**NS: Salinity**	**Gametes**	**n**	**Fertilization**	**n**
9	9.05 ± 0.07	5	9.02 ± 0.08	4
17	17.50 ± 0.22	5	17.04 ± 0.11	4
21	20.63 ± 0.12	5	21.15 ± 0.24	4
30	30.12 ± 0.15	5	30.20 ± 0.32	4
35	34.76 ± 0.21	5	34.68 ± 0.28	4

BS: Temperature	Gametes and fertilization media		
	Beginning (°C)	End (°C)	n
5 °C	4.93 ± 0.02	4.97 ± 0.02	6
10 °C	9.97 ± 0.02	9.93 ± 0.05	6
15 °C	15.03 ± 0.02	15.05 ± 0.02	6
20 °C	20 ± 0	20 ± 0	6
25 °C	24.90 ± 0.03	24.92 ± 0.03	6

NS: Temperature	Gametes and fertilization media		
	Beginning (°C)	End (°C)	n
5 °C	5.04 ± 0.02	5.02 ± 0.02	5
10 °C	10 ± 0.07	9.94 ± 0.04	5
15 °C	15.02 ± 0.04	15.02 ± 0.02	5
20 °C	20.02 ± 0.02	20 ± 0	5
25 °C	24.96 ± 0.02	24.98 ± 0.02	5

Table S2. Sperm concentration of male mussel *M. edulis* individuals before and after centrifugation for sperm mitochondrial function measurements. Raw indicates the raw sperm concentration (10^7 cells ml^{-1}) before centrifugation and stock represents the sperm concentration (10^9 cells ml^{-1}) after centrifugation.

Treatments	15 °C×630 mOsm		15 °C×510 mOsm		15 °C×390 mOsm		15 °C×270 mOsm		15 °C×150 mOsm	
	Raw	Stock	Raw	Stock	Raw	Stock	Raw	Stock	Raw	Stock
N1	5	5.23	8.4	3.4	4.8	2.4	5.3	2.2	5.2	1.3
N2	7.5	4.52	11.5	3.8	5.3	2.8	4.4	4.5	6.9	3.9
N3	7.75	5.5	12.3	4.3	14.5	7.2	4	1.6	22.7	3.8
N4	10.1	8.5	23.4	7.3	6.9	4.7	17.9	2.2	5.5	5
N5	7.1	3.1	5.7	2.1	5.5	2.85	10.9	2	14.6	4.8
N6	3.9	2.4			4.9	2.8				
**Treatments**	**20 °C×630 mOsm**		**20 °C×510 mOsm**		**20 °C×390 mOsm**		**20 °C×270 mOsm**		**20 °C×150 mOsm**	
	Raw	Stock	Raw	Stock	Raw	Stock	Raw	Stock	Raw	Stock
N1	10.2	4.4	3.1	1.9	16.8	8.4	9	5.25	12.5	3.85
N2	5.6	2.75	5.1	4.4	12.6	7	7.7	4.05	9.6	4
N3	5.4	3.5	5	3.95	4.5	2.4	6.3	1.9	21.2	6.85
N4	9.5	4.35	6.5	4.1	5.8	4.05	7.3	5.75	8.3	2.6
N5	3.7	2.8	8.3	4.5	23	3.45	7.9	3.2	16	3.3
N6	10.5	3.6	4.5	3.4	19.9	3.3	6.5	2.15	6.8	2.75

Journal of Experimental Biology • Supplementary information

N7	7.2	4.5	8.4	4.1			10.1	3.8	5.2	2.45
N8	3.8	2.85							6.5	3.75

Treatments	25 °C×630 mOsm		25 °C×510 mOsm		25 °C×390 mOsm		25 °C×270 mOsm		25 °C×150 mOsm	
	Raw	Stock	Raw	Stock	Raw	Stock	Raw	Stock	Raw	Stock
N1	6.7	3.5	3.4	2.6	1.8	2.05	6.1	3.9	7.3	6.1
N2	5.5	3.3	2	1.5	2.5	1.9	10.9	9.4	6.6	3.55
N3	7	4.55	2.4	2	6.1	2.9	5.8	3.7	3.2	3.9
N4	8.5	6.4	10.2	6.6	2.7	3.2	6.1	3.55	3.3	2.5
N5	19.4	5.8	3.4	2.75	5	3.65	3.7	2.8	3.6	4.55
N6	18.4	6.75			4.2	3.3	4.3	4.7	8.8	6.6

Journal of Experimental Biology • Supplementary information

Table S3. Chemical compositions in the assay buffer for distinct sperm mitochondrial exposure.

Assay buffers	Salinity of 5	Salinity of 9	Salinity of 13	Salinity of 17	Salinity of 21
Osmolarity	150 mOsm	270 mOsm	390 mOsm	510 mOsm	630 mOsm
Sucrose	0	0	47mM	167mM	287mM
KCl	43.5mM	103.5mM	130mM	130mM	130mM
NaCl	0	0	10mM	10mM	10mM
HEPES	30mM	30mM	30mM	30mM	30mM
Glucose	10mM	10mM	10mM	10mM	10mM
$MgCl_2$	1mM	1mM	1mM	1mM	1mM
KH_2PO_4	10mM	10mM	10mM	10mM	10mM
BSA	1%	1%	1%	1%	1%
pH	7.2	7.2	7.2	7.2	7.2

Table S4. Significant difference between the treatments on the embryonic development of both mussel populations under distinctive salinity and temperature conditions. *p* values marked in red were only recorded when the significant difference occurred.

BS-Salinity	Unfertilized egg stage		First polar body stage		Two-cell stage		Four-cell stage		Eight-cell stage	
	Treatments Comparison	*p*	Treatments Comparison	*p*	Treatments Comparison	*p*	Treatments Comparison	*p*	Treatments Comparison	*p*
	5 vs 9	<0.001			5 vs 21	0.019	5 vs 13	0.034	5 vs 13	0.012
	5 vs 13	<0.001					5 vs 17	0.044	5 vs 17	0.015
	5 vs 17	<0.001								
	5 vs 21	<0.001								
	13 vs 21	0.021								
	17 vs 21	0.04								
BS-Temperature	Unfertilized egg stage		First polar body stage		Two-cell stage		Four-cell stage		Eight-cell stage	
	Treatments Comparison	*p*	Treatments Comparison	*p*	Treatments Comparison	*p*	Treatments Comparison	*p*	Treatments Comparison	*p*
	5 vs 15 (°C)	0.004	5 vs 10 (°C)	0.012	5 vs 15 (°C)	0.046	5 vs 15 (°C)	0.001	5 vs 20 (°C)	0.002
	5 vs 20 (°C)	0.003			5 vs 25 (°C)	0.02	10 vs 15 (°C)	0.024	5 vs 25 (°C)	0.025
									10 vs 20 (°C)	0.002
									10 vs 25 (°C)	0.025

Journal of Experimental Biology • Supplementary information

									15 vs 20 (°C)	0.003
NS-Salinity	Unfertilized egg stage		First polar body stage		Two-cell stage		Four-cell stage		Eight-cell stage	
	Treatments Comparison	*p*	Treatments Comparison	*p*	Treatments Comparison	*p*	Treatments Comparison	*p*	Treatments Comparison	*p*
	9 vs 21	0.009	9 vs 17	0.004	9 vs 17	0.034	9 vs 21	0.001	9 vs 30	0.025
	9 vs 30	0.002			9 vs 21	0.006	9 vs 30	<0.001	9 vs 35	0.017
							9 vs 35	0.001	17 vs 30	0.025
							17 vs 30	0.046	17 vs 35	0.017
NS-Temperature	Unfertilized egg stage		First polar body stage		Two-cell stage		Four-cell stage		Eight-cell stage	
	Treatments Comparison	*p*	Treatments Comparison	*p*	Treatments Comparison	*p*	Treatments Comparison	*p*	Treatments Comparison	*p*
	5 vs 15 (°C)	0.015					5 vs 15 (°C)	0.003	5 vs 25 (°C)	0.013
	5 vs 20 (°C)	0.014								
	5 vs 25 (°C)	0.022								

Table S5. Two-way repeated measures ANOVA of the exposure time and salinity/temperature on sperm motility and velocity of mussels *M. edulis* from the Baltic and North Sea. Only data following the normal distribution are presented.

**VAP**		BS-Salinity	
Source	df	*F*	*p*
Exposure time	1	60.024	<0.001
Exposure time * Salinity	4	1.159	0.358
**Motility rate**		BS-Temperature	
Source	df	*F*	*p*
Exposure time	1	181.474	<0.001
Exposure time * Temperature	4	3.12	0.033
**VCL**		BS-Temperature	
Source	df	*F*	*p*
Exposure time	1	58.921	<0.001
Exposure time * Temperature	4	1.068	0.393
**VAP**		BS-Temperature	
Source	df	*F*	*p*
Exposure time	1	145.351	<0.001
Exposure time * Temperature	4	2.556	0.064

**VAP**		NS-Salinity	
Source	df	*F*	*p*
Exposure time	1	15.191	0.001
Exposure time * Salinity	4	0.898	0.484
**Motility rate**		NS-Temperature	
Source	df	*F*	*p*
Exposure time	1	202.061	<0.001
Exposure time * Temperature	4	7.09	0.001
**VCL**		NS-Temperature	
Source	df	*F*	*p*
Exposure time	1	118.027	<0.001
Exposure time * Temperature	4	1.75	0.179

Journal of Experimental Biology • Supplementary information
